# Hyperaminoacidemia from interrupted glucagon signaling increases pancreatic acinar cell proliferation and size via mTORC1 and YAP pathways

**DOI:** 10.1016/j.isci.2024.111447

**Published:** 2024-11-22

**Authors:** Chunhua Dai, Yue Zhang, Yulong Gong, Amber Bradley, Zihan Tang, Katelyn Sellick, Shristi Shrestha, Erick Spears, Brittney A. Covington, Jade Stanley, Regina Jenkins, Tiffany M. Richardson, Rebekah A. Brantley, Katie Coate, Diane C. Saunders, Jordan J. Wright, Marcela Brissova, E. Danielle Dean, Alvin C. Powers, Wenbiao Chen

**Affiliations:** 1 Division of Diabetes, Endocrinology and Metabolism, Department of Medicine, Vanderbilt University Medical Center, Nashville, TN, USA; 2 Department of Molecular Physiology & Biophysics, Vanderbilt University, Nashville, TN, USA; 3 VA Tennessee Valley Healthcare System, Nashville, TN, USA

**Keywords:** Biomolecules, Molecular biology, Cell biology, Model organism

## Abstract

Increased blood amino acid levels (hyperaminoacidemia) stimulate pancreas expansion by unclear mechanisms. Here, by genetic and pharmacological disruption of glucagon receptor (GCGR) in mice and zebrafish, we found that the ensuing hyperaminoacidemia promotes pancreatic acinar cell proliferation and cell hypertrophy, which can be mitigated by a low protein diet in mice. In addition to mammalian target of rapamycin complex 1 (mTORC1) signaling, acinar cell proliferation required *slc38a5*, the most highly expressed amino acid transporter gene in both species. Transcriptomics data revealed the activation signature of yes-associated protein (YAP) in acinar cells of mice with hyperaminoacidemia, consistent with the observed increase in YAP-expressing acinar cells. Yap1 activation also occurred in acinar cells in *gcgr*−/− zebrafish, which was reversed by rapamycin. Knocking down *yap1* in *gcgr*−/− zebrafish decreased mTORC1 activity and acinar cell proliferation and hypertrophy. Thus, the study discovered a previously unrecognized role of the YAP/Taz pathway in hyperaminoacidemia-induced acinar cell hypertrophy and hyperplasia.

## Highlights

• Hyperaminoacidemia causes acinar cell hypertrophy and hyperplasia in mice and zebrafish
• Zebrafish acinar cell hypertrophy and proliferation require *slc38a5b*
• Hyperaminoacidemia activates YAP/TAZ in acinar cells
• Zebrafish mTORC1 activation and acinar cell hypertrophy and proliferation entail *yap1*

## Graphical abstract

**Figure:**
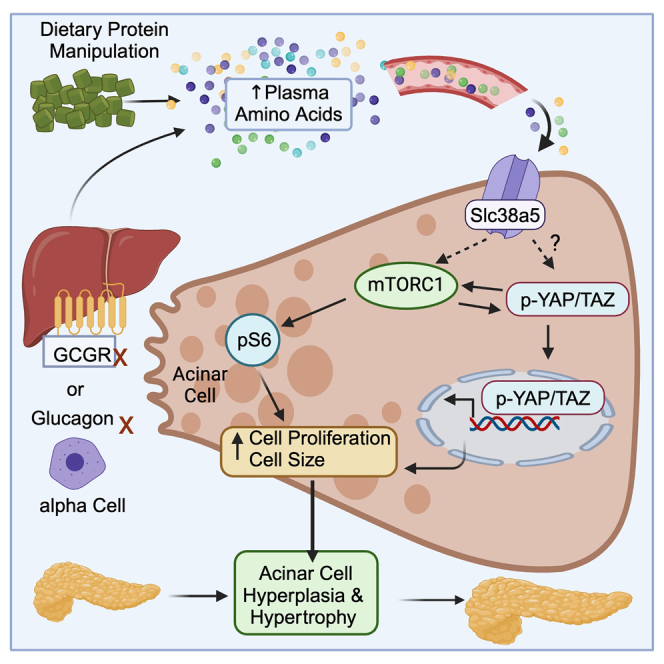

## Introduction

Acinar cells are the most abundant cell type of the pancreas, constituting 70% of the cell number and 85% of the pancreas mass.^1^^,^^2^^,^^3^ They produce, store, and secrete large amounts of digestive enzymes necessary for the digestion of proteins, carbohydrates, and fats.^4^^,^^5^ Although pancreas mass is stable in adults, it can be changed by diet protein levels by unclear mechanisms.

Pancreas mass changes according to the levels of protein intake. Chronic protein deficiency causes pancreas atrophy both in humans and animal models.^6^^,^^7^ Conversely, a high-protein diet induces pancreas expansion in animal models.^8^^,^^9^ Both acinar cell hypertrophy and hyperplasia contribute to pancreas expansion.^8^ In mice, increased levels of blood amino acids (hyperaminoacidemia) and cholecystokinin (CCK) from ingestion of a high protein diet induce acinar cell proliferation and hypertrophy through activation of the mammalian target of rapamycin complex 1 (mTORC1).^10^^,^^11^ Whether other pathways are also necessary for hyperaminoacidemia-induced pancreas growth is unknown.

Interrupted glucagon signaling (IGS) also results in hyperaminoacidemia^12^^,^^13^^,^^14^^,^^15^^,^^16^ and increased pancreas mass.^14^^,^^17^^,^^18^^,^^19^ Hyperaminoacidemia results from decreased glucagon-stimulated amino acid (aa) catabolism through ureagenesis and gluconeogenesis in the liver.^15^^,^^16^^,^^20^ Unlike diet-induced hyperaminoacidemia, IGS-induced hyperaminoacidemia persists during fasting and is decoupled from enteroendocrine secretion. Nevertheless, the total pancreas mass of *Gcgr*−/− mice and mice with liver-specific GCGR deletion is 1.5–3.5 times larger than that of control littermates by 6 weeks of age.^17^^,^^18^^,^^19^ Patients with Mahvash disease, an autosomal recessive condition of biallelic *GCGR* loss of function, also have hyperaminoacidemia and an enlarged pancreas.^18^^,^^21^^,^^22^^,^^23^^,^^24^ Although IGS causes profound alpha cell hypertrophy, hyperplasia, and glucagonoma,^16^^,^^17^^,^^18^^,^^19^^,^^20^ the pancreas expansion is unlikely the sole result of increased endocrine cells since endocrine mass makes up less than 2% of total pancreas mass. Expansion of the exocrine pancreas is likely the major contributor.

To investigate the cellular and molecular mechanisms of hyperaminoacidemia-induced pancreas expansion, we used multiple models of IGS in mice and zebrafish. We used a low protein diet (LPD) to correct hyperaminoacidemia in an IGS mouse model. We demonstrated that IGS induces acinar cell hyperplasia and hypertrophy, which can be blunted by lowering blood aa levels with a LPD. Both acinar cell hyperplasia and hypertrophy require mTORC1. Transcriptomic analysis of acinar cells revealed *Slc38a5* as the most highly expressed aa transporter and provided evidence for activation of yes-associated protein (YAP) signaling in IGS mice. Knockdown experiments in *gcgr*−/− zebrafish demonstrated an essential role for *slc38a5b* and *yap*1 in acinar cell hyperplasia and hypertrophy. These results uncovered multiple mechanisms of hyperaminoacidemia-induced pancreas expansion and revealed a previously unappreciated role for SLC38A5 and the YAP/TAZ pathway in mediating aa-induced acinar cell proliferation and hypertrophy.

## Results

### IGS causes both hyperplasia and hypertrophy of acinar cells

Pancreas expansion is a prominent phenotype in *Gcgr*−/− mice and patients with biallelic inactivating mutations in *GCGR*. To assess the mechanisms of pancreas expansion under IGS, we characterized pancreas size in two additional mouse models and one zebrafish model in which pancreas expansion has not been previously investigated. The first mouse model is *Gcg*−/− mice that lack the glucagon-encoding exon of the preproglucagon gene on the immunodeficient NOD scid gamma (NSG) background (Figure S1A).^25^ Compared to control, *Gcg*−/− mice exhibited lower blood glucose levels (Figure S1B), more than 2-fold increase of total serum amino acids (Figure S1C), and no change in body weight (Figure S1D). They also had increased absolute and relative pancreas weight (Figures S1E and S1F). The second mouse model is GCGR-Ab treated C57BL/6J mice (Figure S2A). Compared to IgG treatment, GCGR-Ab treatment for 8 weeks resulted in a more than 2-fold increase of serum amino acids, as reported previously (Figure S2B).^15^^,^^20^ At 2, 4, and 8 weeks of treatment, GCGR-Ab-treated mice had significantly decreased blood glucose levels (Figures S2C, S2G, and S2K), normal body weight (Figures S2D, S2H, and S2L), and a progressive increase absolute pancreas weight (Figures S2E, S2I, and S2M) and relative pancreas weight (Figures S2F, S2J, and S2N). We also compared individual serum amino acids in mice treated for 8 weeks. Except for tryptophan, phenylalanine, and cysteine, all other amino acids were increased by GCGR-Ab treatment (Figures S3A–S3C), confirming our previous results.^20^ To determine whether pancreas expansion is conserved across species, we evaluated pancreas size in *gcgr*−/− fish carrying *Tg*(*ela3l:EGFP*) (Figure S4A), which has hyperaminoacidemia at both larval and adult stages.^26^^,^^27^ Both the pancreas volume and pancreas area were significantly greater at 18 dpf compared to controls (Figures S4B–S4D). These data demonstrated that IGS in all models induces pancreas expansion.

**Figure 1:**
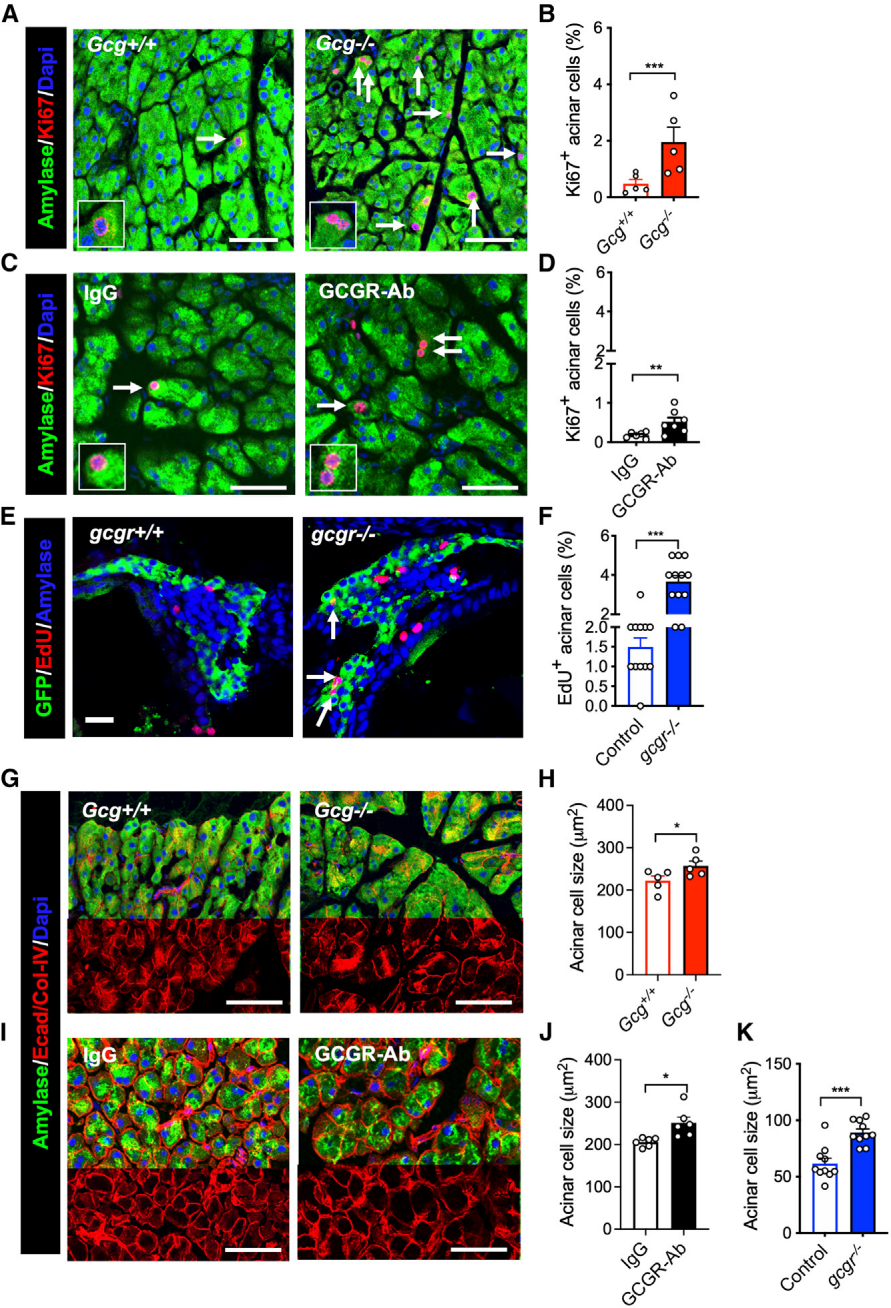
IGS increases acinar cell proliferation and cell size (A and C) Representative images of acinar tissue immunofluorescence of amylase (green) and Ki67 (red). DAPI (blue) was used to label the nuclei. The pancreas sections were from *Gcg*+/+ or *Gcg*−/− mice (A) or from C57BL/J6 mice treated with IgG or GCGR-Ab (C). (B and D) Quantification of Ki67 positive acinar cell (n = 5–7). Arrows point to Ki67+ cells. (E) Representative immunofluorescent images of pancreas sections from 18 dpf zebrafish. Green (GFP), red (EdU), and blue (Amylase). Arrows, EdU+ acinar cells. (F) Quantification of EdU-positive acinar cells (*n* = 12). (G and I) Representative images of acinar tissue immunofluorescence of Amylase (Green), E-cadherin, and Collagen (Red). DAPI (blue) was used to label the nuclei. The pancreas sections were from *Gcg*+/+ or *Gcg*−/− mice (G) or from C57BL/J6 mice treated with IgG or GCGR-Ab (I). (H and J) Measurements of acinar cell size (by area) in the two mouse models. (K) Average acinar cell size of control and *gcgr*−/− zebrafish. Each data point is the average of more than 50 cells from one fish. Scale bar, 15 μm in (E). Scale bar, 50 μm in others. Data are represented as mean ± SEM. ∗*p* < 0.05, ∗∗*p* < 0.01, ∗∗∗*p* < 0.001. Student’s t test.

Pancreas expansion could result from increased acinar cell size, cell proliferation, or both. Since acinar cells are the major cell type of the pancreas,^1^^,^^2^ we reasoned that endocrine expansion could not be responsible for the increased pancreas size. We did not observe signs of ductal expansion and did not see signs of pancreas edema on examination of the pancreas or in pancreatic sections. So, we determined acinar cell proliferation, size, and apoptosis. We found that *Gcg−/−* and GCGR-Ab treated mice, and *gcgr*−/− zebrafish significantly increased the percentage of proliferating acinar cells (Ki67+ in mice, EdU+ in zebrafish) compared to their controls (Figures 1A–1F). These data indicate that IGS stimulates acinar cell proliferation in mice and zebrafish.

We next compared acinar cell size between IGS and control animals. Compared to the control, the average acinar cell size was larger in *Gcg*−/− mice (Figures 1G and 1H) and GCGR-Ab treated mice (Figures 1I and 1J). As peri-islet acinar cells are more hypertrophic and proliferative,^28^^,^^29^ we measured acinar cell size in different regions and found increased acinar cell size throughout the pancreas (Figure S5). Increased acinar cell size was also observed in *gcgr*−/− zebrafish (Figure 1K). These data indicate that IGS induces acinar cell hypertrophy in mice and zebrafish. TUNEL assay did not find a change in acinar cell apoptosis in GCGR-Ab treated mice (Figures S6A and S6B). Taken together, these results demonstrated that IGS induces adaptive proliferation and hypertrophy in acinar cells.

### IGS-induced pancreas expansion is independent of GLP-1 and pancreatitis, but requires hyperaminoacidemia

**Figure 2:**
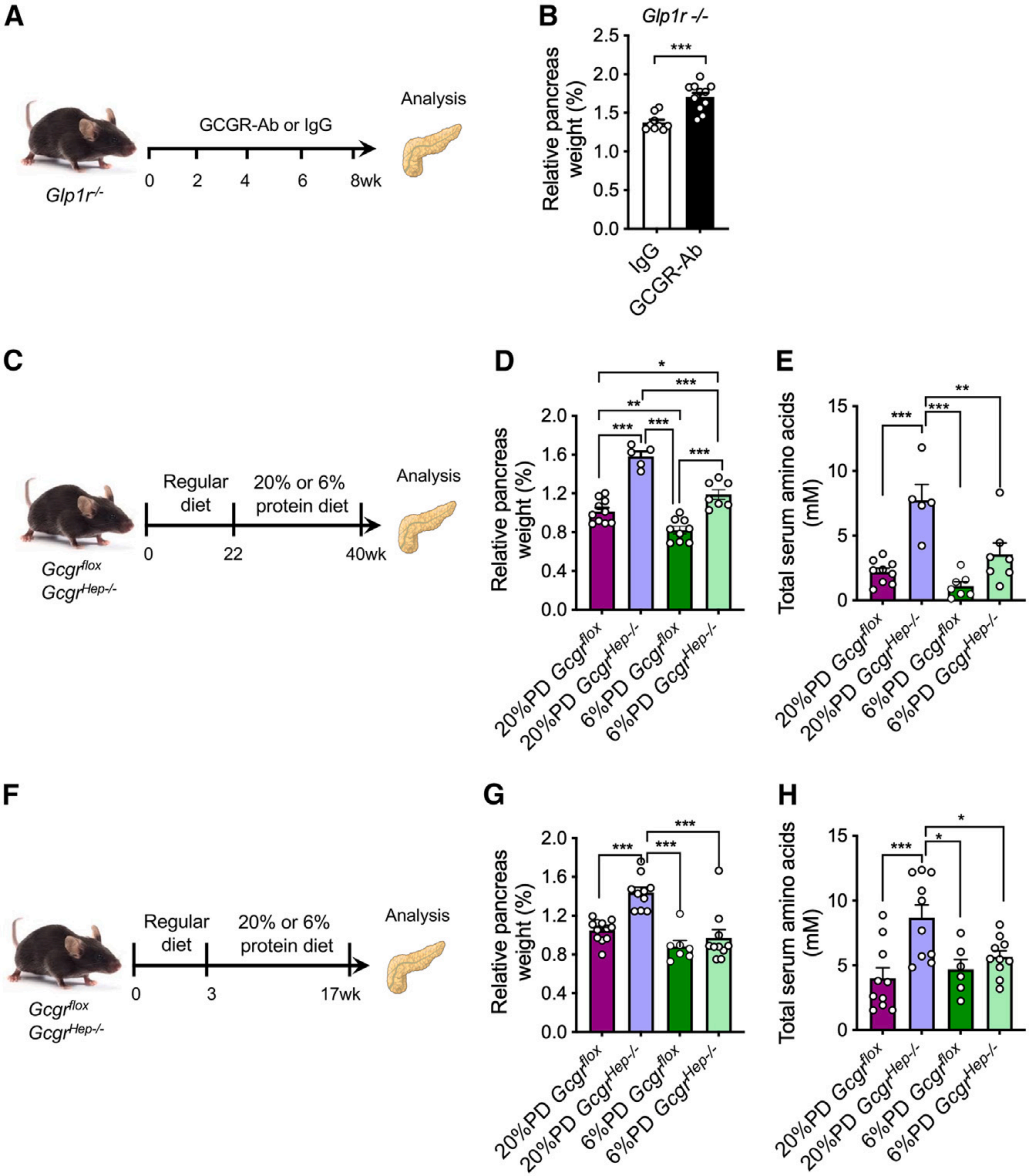
Hyperaminoacidemia, but not GLP-1, contributes to the increased pancreas mass in the GCGR-Ab-treated mice (A) Schematic of experimental design in *Glp1r*−/− and control mice. (B) Pancreas mass in *Glp1r*−/− mice treated with IgG or GCGR-Ab (n = 9–11/treatment). ∗∗∗*p* < 0.001. Student’s t test. (C) Experimental outline depicting the treatment of IGS-induced pancreas expansion by a low protein diet. (D) Relative pancreas weight in *Gcgr*^*hep*^*−/−* and control mice on 20% or 6% protein diet. (E) Total blood aa in *Gcgr*^*hep*^*−/−* and control mice on 20% or 6% protein diet. (F) Experimental outline to prevent IGS-induced pancreas expansion. (G) Relative pancreas weight in *Gcgr*^*hep*^*−/−* and control mice on 20% or 6% protein diet. (H) Total blood aa in *Gcgr*^*hep*^*−/−* and control mice on 20% or 6% protein diet. Data are represented as mean ± SEM. ∗*p* < 0.05, ∗∗*p* < 0.01, ∗∗∗*p* < 0.001. One-way ANOVA followed by Turkey’s multiple comparisons tests.

Pharmacological activation of glucagon-like peptide-1 receptor (GLP-1R) has been shown to promote acinar cell growth and proliferation.^30^^,^^31^ As *Gcgr*−/− mice have increased α cell mass and serum GLP-1 levels,^17^^,^^25^^,^^32^ we evaluated whether GCGR-Ab treatment increases GLP-1/GLP-1R signaling in C57BL/J6 mice. We found that an 8-week GCGR-Ab treatment did not increase *Glp1r* mRNA in acinar cells compared to IgG treatment (Figure S7A). To determine whether GLP1R is necessary for IGS-induced pancreas expansion, we treated *Glp1r*−/− mice with GCGR-Ab or IgG for 8 weeks (Figure 2A). The treatment decreased blood glucose as expected (Figure S7B), but still induced pancreas expansion compared to IgG treatment (Figures 2B, S7C, and S7D). These results indicate that the GLP-1 pathway is unnecessary for IGS-induced pancreas expansion.

Pancreatitis has been reported to induce acinar cell proliferation.^33^ We, therefore, examined the presence of immune cells in the pancreas using CD45 immunofluorescence. There was no increase of CD45^+^ immune cells in the exocrine tissue of GCGR-Ab treated mice (Figures S7E and S7F). Furthermore, there was no change in serum CCK levels or expression of either the major CCK receptor *Cckar* or the minor CCK receptor *Cckbr* in acinar cells in GCGR-Ab treated mice compared to IgG-treated control (Figures S7G–S7I).

As hyperaminoacidemia from a protein-rich diet causes pancreas expansion, we assessed the role of hyperaminoacidemia in IGS-induced pancreas expansion. We decreased blood aa levels through diet in a fourth IGS model, hepatocyte-specific deletion of *Gcgr* (*Gcgr*^*hep−/−*^ mice), with *Gcgr*^*flox*^ mice as control.^19^ As dietary protein is the major contributor to blood aa,^34^ we changed the diet of 22-week-old *Gcgr*^*hep−/−*^ and *Gcgr*^*flox*^ mice from a chow diet to a regular protein diet (RPD) or a LPD for 18 weeks (Figure 2D). *Gcgr*^*hep−/−*^ and *Gcgr*^*flox*^ mice did not have a significant difference in body weight on the same diet, but *Gcgr*^*flox*^ mice on the RPD weighed more than both *Gcgr*^*hep−/−*^ and *Gcgr*^*flox*^ mice on the LPD (Figure S8A). On the same diet, *Gcgr*^*hep−/−*^ mice had a larger relative pancreas weight (Figures 2D and S8B) and more than 3-fold higher total plasma aa levels than *Gcgr*^*flox*^ mice (Figure 2E). With the same genotype, mice had higher total plasma aa levels on the RPD than on the LPD (Figure 2E). However, the relative pancreas weight of *Gcgr*^*hep−/−*^ mice on the LPD was still higher than those in *Gcgr*^*flox*^ mice on the RPD (Figures 2D and S8B). Therefore, LPD only partially reduced pancreas expansion in *Gcgr*^*hep−/−*^ mice.

Some of the pancreas weight increase in *Gcgr*^*hep−/−*^ mice may have occurred before the diet switch. To test this, we started feeding *Gcgr*^*hep−/−*^ and *Gcgr*^*flox*^ mice the RPD or the LPD at weaning for 14 weeks (Figure 2F). Mice on LPD had a small reduction of body weight and markedly smaller absolute pancreas weight than those on the RPD (Figures S8C and S8D). As expected, on the RPD the relative pancreas weight and total blood aa levels in *Gcgr*^*hep−/−*^ mice were higher than those of *Gcgr*^*flox*^ mice (Figures 2G and 2H). Strikingly, there was no difference in relative pancreas weight or total blood aa levels between *Gcgr*^*hep−/−*^ and *Gcgr*^*flox*^ mice on the LPD, both were similar to *Gcgr*^*flox*^ mice on the RPD (Figures 2G and 2H). Therefore, we conclude that hyperaminoacidemia is essential for IGS-induced pancreas expansion.

### SLC38A5 mediates hyperaminoacidemia-induced acinar cell hyperplasia and pancreas expansion

**Figure 3:**
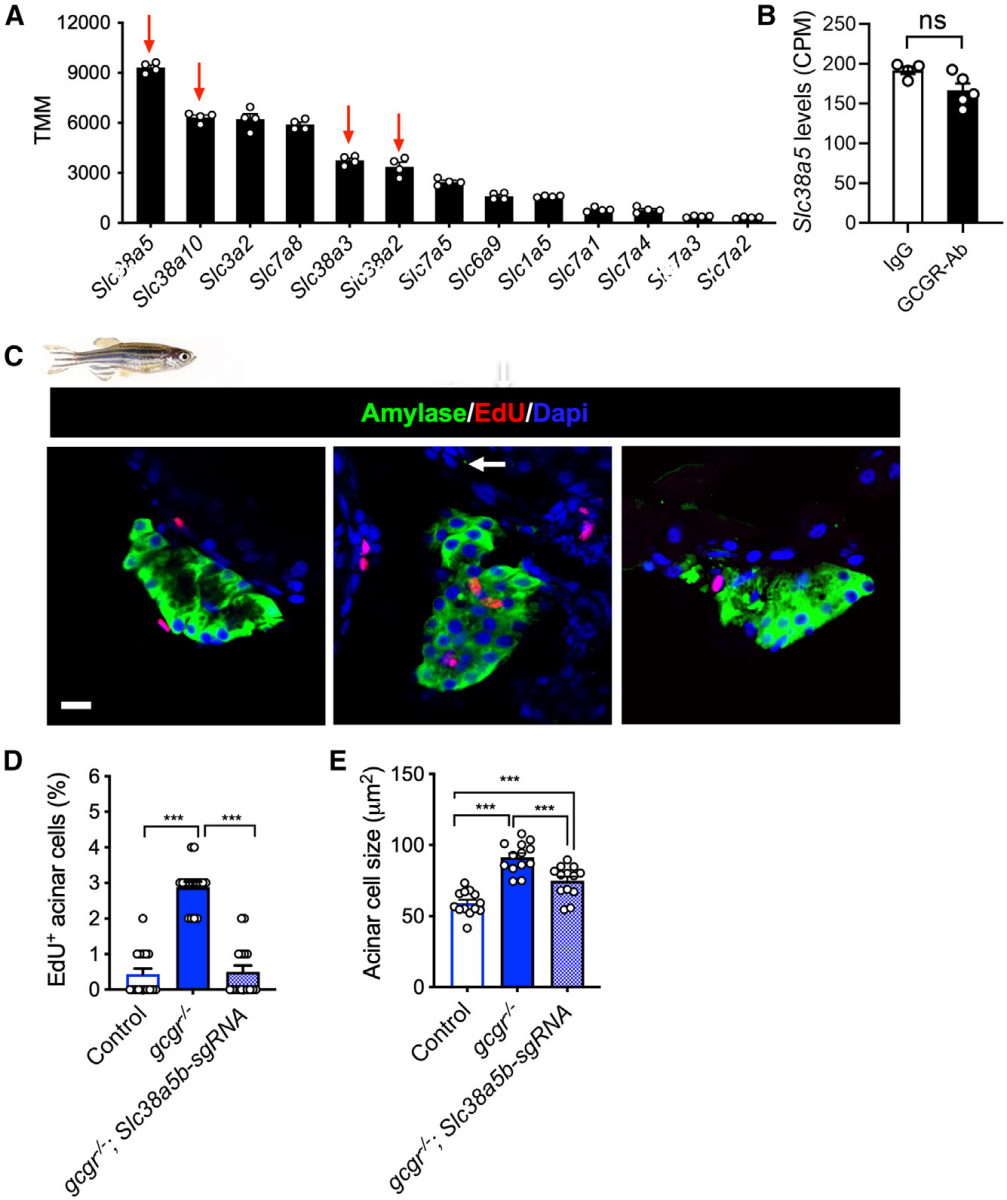
Slc38a5 mediates acinar cell growth (A) The top 11 amino acid transporters expressed in mouse acinar cells (*n* = 5, data from RNA-seq). (B) Slc38a5 expression in acinar cells of IgG and GCGR-Ab treated mice. CPM, count per million. Student’s t test. (C) Representative images of zebrafish pancreas from control, *gcgr*−/−, and *gcgr*−/− with *slc38a5b* knockdown groups. Green, amylase; red, EdU; blue, DAPI. Scale bar, 10 μm. (D) Quantification of EdU+ acinar cells in control, *gcgr*−/−, and *gcgr*−/− with *slc38a5b* knockdown group (*n* = 16/genotype). (E) Acinar cell size in control, *gcgr*−/−, and *gcgr*−/− with *slc38a5b* knockdown groups. Each data point is the average of more than 50 cells from the same fish. Data are represented as mean ± SEM. ∗*p* < 0.05, ∗∗*p* < 0.01, ∗∗∗*p* < 0.001. One-way ANOVA followed by Turkey’s Multiple Comparisons Test.

Extracellular amino acids are transported intracellularly by aa transporters (aaTs) to exert their functions. Consistent with the expression pattern in human acinar cells,^35^ our acinar cell RNA-seq data indicated that *Slc38a5,* encoding a neutral aa transporter, was the most highly expressed aaT in mouse acinar cells (Figure 3A). Its expression was not changed by GCGR-Ab treatment (Figure 3B). To determine whether *Slc38a5* is necessary for the observed acinar cell growth and proliferation, we used CRISPR/Cas9 to knock down the zebrafish ortholog *slc38a5b*, the second most highly expressed aaT in acinar cells (Table S1).^36^^,^^37^ Knockdown of *slc38a5b* with 2 efficient sgRNAs in zebrafish decreased the proliferation and size of acinar cells in *gcgr−/−* zebrafish (Figures 3C–3E) (Table S2), suggesting that Slc38a5b is crucial for IGS-induced acinar hyperplasia and hypertrophy.

### Activation of mTORC1 and YAP/TAZ contributes to hyperaminoacidemia-induced acinar cell hyperplasia and pancreas expansion

**Figure 4:**
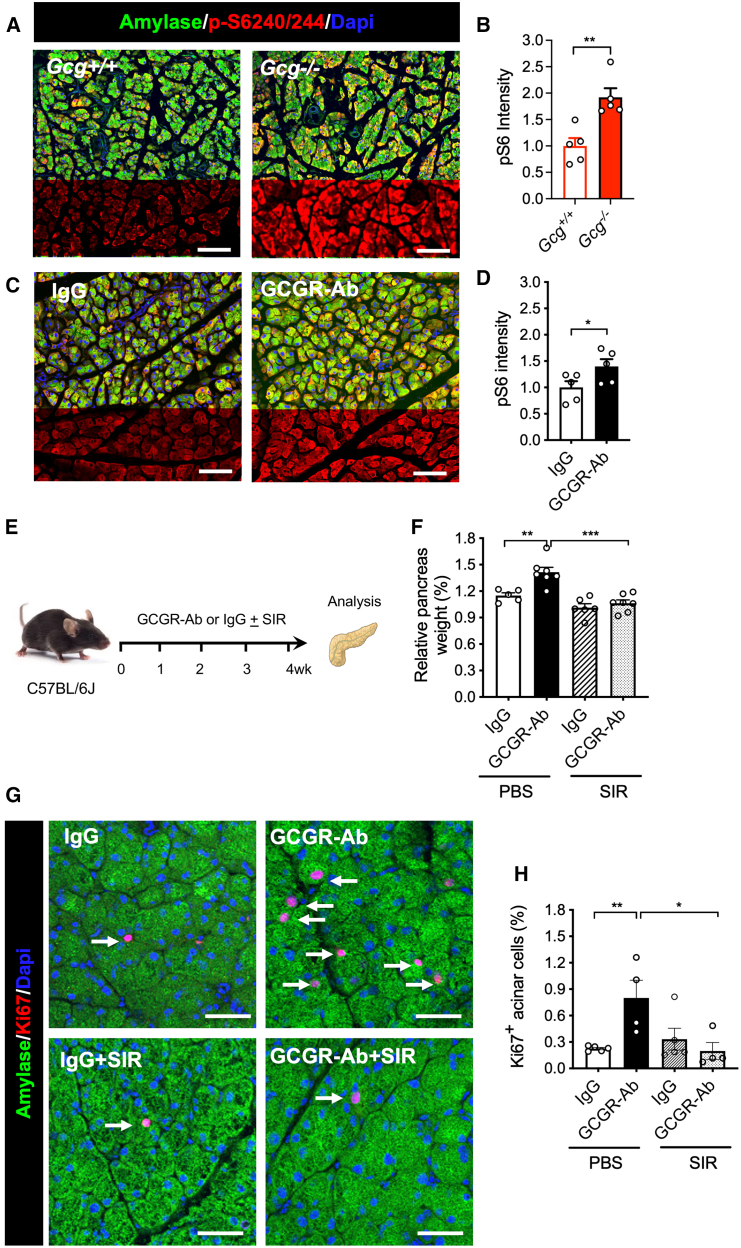
IGS activates mTORC1 pathway in acinar cells (A and C) Representative images of pancreas immunofluorescence from the 2 mouse models. Green, amylase; red, phosphor-S6 (240/244); blue, DAPI. (B and D) Quantification of pS6 intensity in *Gcg*−/− mice (B) or antibody-treated mice (D) and their controls (*n* = 5/group). The intensity was normalized to control mice or the IgG treatment group. ∗*p* < 0.05, ∗∗*p* < 0.01, Student’s t test. (E) Schematic experimental design for treating mice with sirolimus (rapamycin) treatment. (F) Relative pancreas weight in the four groups (n = 5–6/group). (G) Representative immunofluorescence images of acinar tissues. Amylase, green; Ki67, red; DAPI, blue. Arrows point to Ki67+ acinar cells. (H) Quantification of Ki67+ acinar cells in the four groups (*n* = 5/group). Scale bar, 100 μm (A and C), 50 μm (G). Data are represented as mean ± SEM. ∗*p* < 0.05, ∗∗*p* < 0.01, ∗∗∗*p* < 0.001. One-way ANOVA followed by Turkey’s Multiple Comparisons Test.

AAs are effective activators of mTORC1 pathway, an important signaling pathway for cell growth and proliferation.^38^^,^^39^ The levels of serine 240/244 in S6 ribosomal protein (pS6), an indicator of mTORC1 activity, were significantly increased in *Gcg*−/− mice and GCGR-Ab treated mice (Figures 4A–4D), indicating mTORC1 activation in acinar cells. Treatment with IgG in the presence or absence of mTORC1 inhibitor sirolimus (rapamycin) for 4 weeks significantly increased blood glucose levels as expected (Figures 4E and S9D),^40^^,^^41^ and did not impact body weight (Figure S9E). Importantly, sirolimus treatment abolished GCGR-Ab-induced increase in relative pancreas weight (Figures 4F and S9F). Moreover, sirolimus treatment also reduced the percentage of Ki67-positive acinar cells in GCGR-Ab-treated mice (Figures 4G and 4H). These data indicate that mTORC1 activity is required for IGS-induced pancreas expansion and acinar cell proliferation.

**Figure 5:**
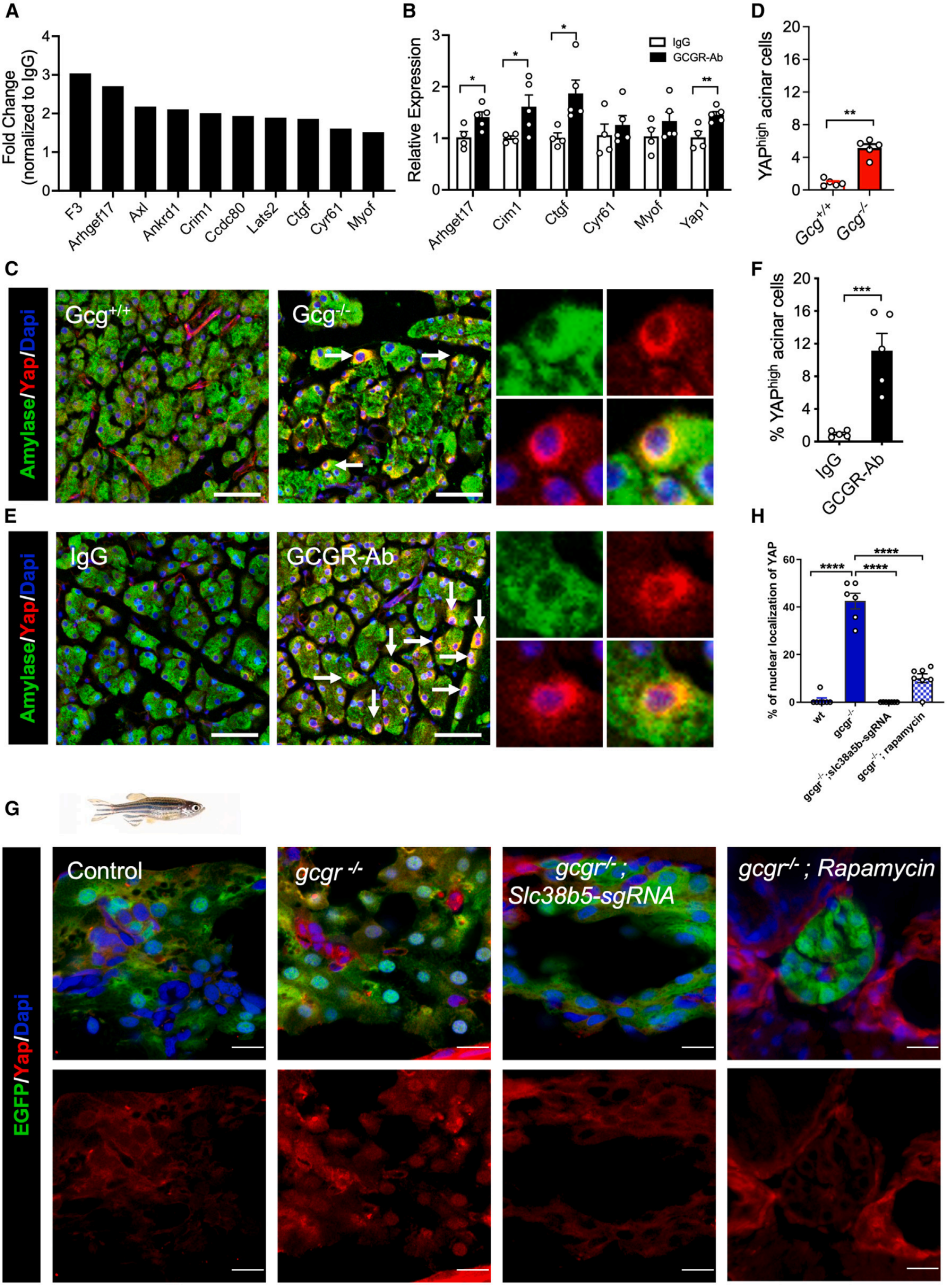
IGS activates the YAP/Taz pathway (A) Upregulation of YAP target genes in acinar cells from GCGR-Ab treated mice. Data are from RNA-seq. (B) RT-qPCR analysis of selected YAP target genes in mRNA from the pancreas of IgG and GCGR-Ab treated mice (n = 4–5/group, compared IgG vs. GCGR-Ab each gene). (C and E) Representative immunofluorescence images of YAP (red) in acinar cells (amylase, green) in the two mouse models. Arrows point to a high expression of YAP. Scale bar, 50 μm. (D and F) Quantifications of the percentage of acinar cells with high YAP expression (*n* = 5/group). *p* < 0.05, ∗∗*p* < 0.01, ∗∗∗*p* < 0.001 Student’s t test. (G) Representative immunofluorescence images of Yap in pancreas sections of WT, *gcgr*−/−, and *gcgr*−/− with *slc38a5b* knockdown, and *gcgr*−/− with rapamycin treatment. All fish carry *Tg(ela3l:EGFP)* transgene that labels acinar cells with EGFP (scale bar, 10 μm). The EGFP- cells with high Yap1 signal are likely ductal cells. (H) Quantification of the percentage of acinar cell with nuclear Yap1. *n* = 7, 6, and 7 for each group, respectively. Data are represented as mean ± SEM. ∗*p* < 0.05, ∗∗*p* < 0.01, ∗∗∗*p* < 0.001. One-way ANOVA followed by Tukey’s multiple comparisons test.

To examine other pathways that are involved in hyperaminoacidemia-induced acinar cell proliferation, we analyzed RNA-seq data from IgG- and GCGR-Ab-treated mice (Figures S10A–S10E). Ingenuity and gene ontology (GO) term analysis identified numerous upregulated pathways involved in proliferation and cell growth (Figures S10D and S10E). Of note, common YAP/TAZ target genes were upregulated in GCGR-Ab-treated acinar cells (Figure 5A). YAP and TAZ are two paralogs that regulate cell proliferation and organ size in multiple tissues via activation of the TEA domain (TEAD) transcription factors in the nucleus.^38^^,^^42^ They are negatively regulated by multiple mechanisms including phosphorylation-mediated degradation.^42^^,^^43^ In mouse pancreas, YAP expression is restricted in ductal cells as YAP suppression is necessary for normal acinar cell and endocrine cell differentiation and identity.^44^^,^^45^ Nevertheless, YAP has been shown to play a role in injury-induced postnatal acinar cell proliferation.^46^ Of genes whose expression is highly associated with YAP/TAZ activity,^47^ 10 were upregulated by more than 1.5-fold in GCGR-Ab treated acinar cells compared to IgG-treated controls (Figure 5A). Quantitative RT-PCR analysis confirmed that expression of YAP/TAZ target genes *Ctgf*, *Crim1*, and *Arhgef17*, and *Yap1* itself was significantly increased in the acinar cells of GCGR-Ab treated mice (Figure 5B). YAP immunofluorescence showed a 7- and 10-fold increase of YAP-high acinar cells in *Gcg*−/− mice and GCGR-Ab-treated mice over control, respectively (Figures 5C–5F). However, a substantial YAP signal remained cytoplasmic, indicating weak activation. Unlike in mice, *yap1* is expressed in acinar cells in adult zebrafish.^37^ In contrast, *taz* expression is restricted to the ductal cells and absent in acinar cells.^37^ Immunofluorescence indicated that Yap1 was present in most acinar cells of control zebrafish at 18 dpf but rarely in the nucleus (<1%) (Figures 5G and 5H). Nonetheless, Yap1 was primarily nuclear in 42% of acinar cells in *gcgr−/−* fish, an effect that was blocked by CRISPR/Cas9-mediated knockdown of *slc38a5b* and rapamycin (Figures 5G, 5H, and S11). These results indicate that YAP1 is activated in acinar cells by IGS in both mice and zebrafish and its activation requires SLC38A5 and mTORC1.

**Figure 6:**
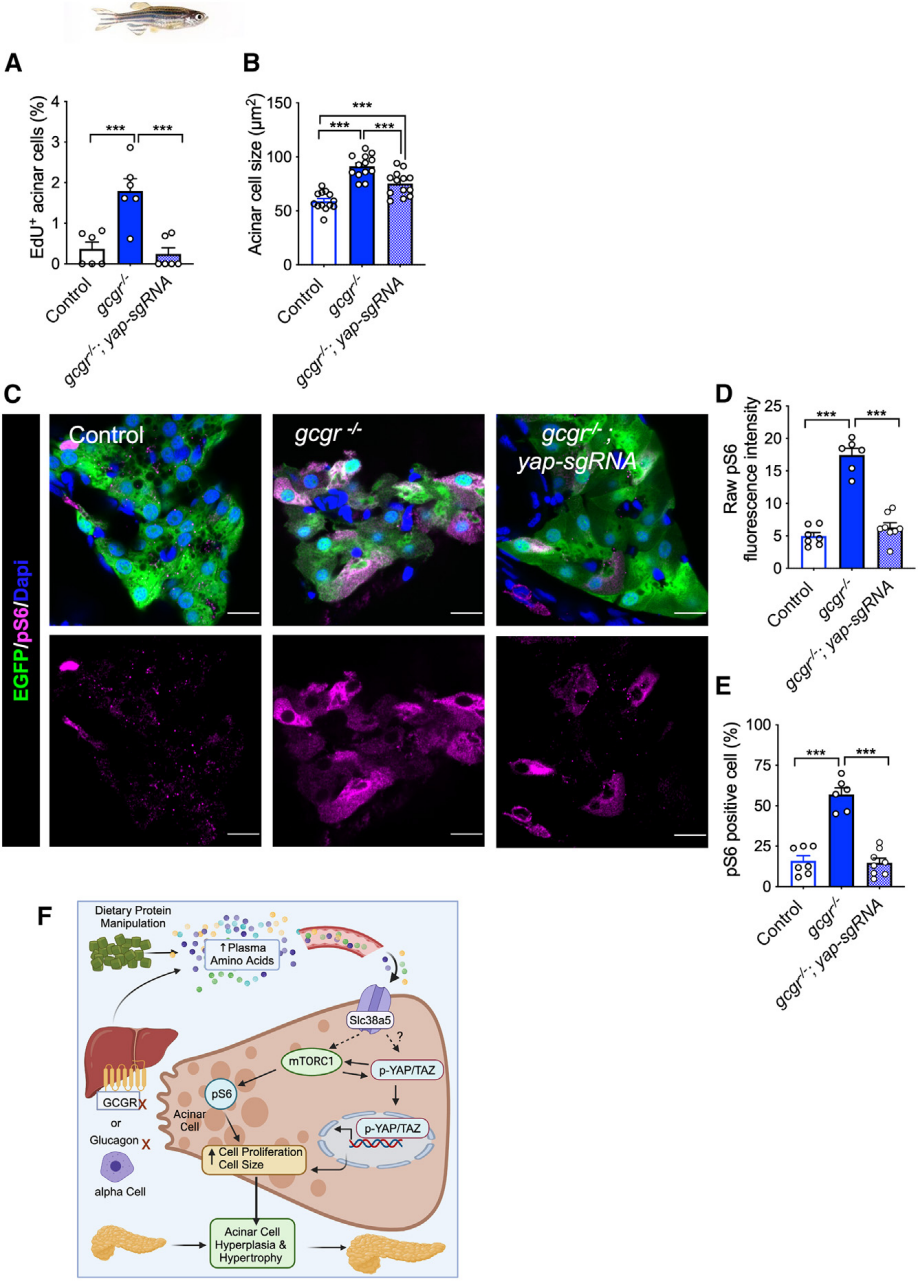
Yap1 is required for mTORC1 activation in *gcgr*−/− fish (A and B) Quantification of the percentage of EdU-labeled acinar cells and the acinar cell size in the pancreas sections WT, *gcgr*−/−, and *gcgr*−/− with *yap1* knockdown. (C) Representative immunofluorescence images of pS6(240/244) in pancreas sections. All fish carry the *Tg(ela3l:EGFP)* transgene that labels acinar cells with EGFP (scale bar, 10 μm). (D) Quantification of raw pS6 signal intensity in acinar cells of these fish. (E) Quantification of the percentage of pS6-positive acinar cells in the pancreas sections. *n* = 7, 6, 8 for each group). Data are represented as mean ± SEM. ∗*p* < 0.05, ∗∗*p* < 0.01, ∗∗∗*p* < 0.001, ∗∗∗∗*p* < 0.0001. One-way ANOVA followed by Tukey’s multiple comparisons test. (F) Proposed model of IGS-induced acinar cell hyperplasia and hypertrophy.

To determine if YAP/TAZ function is necessary for IGS-induced pancreas expansion, we knocked down *yap1* and *taz* individually and in combination in *gcgr*−/− zebrafish using two effective sgRNAs together for each gene (Table S2). Fish with *yap1* and *taz* double knockdown did not survive beyond 14 dpf, consistent with the essential role of these genes in zebrafish development.^48^^,^^49^ Knockdown of *yap1* in *gcgr*−/− fish decreased acinar cell proliferation (Figure 6A) and acinar cell size (Figure 6B). In contrast, the knockdown of *taz* in *gcgr*−/− fish did not affect acinar cell size (Figure S11), consistent with its lack of expression.^37^ As in mice, the levels of acinar cell pS6 were significantly increased in *gcgr−/−* fish. Interestingly, the knockdown of *yap1* in *gcgr*−/− zebrafish reduced pS6 levels to those in WT controls (Figures 6C–6E). Taken together, these results indicate Yap signaling is necessary for mTORC1 activation and IGS-driven acinar cell hyperplasia and hypertrophy.

## Discussion

Pancreas mass varies several fold in humans due to genetic and nutritional differences and diseases. Dietary protein is an important determinant of pancreas size by affecting gastrointestinal hormones and aminoacidemia. High dietary protein induces proliferation and hypertrophy in acinar cells,^8^ which is different from the primarily hypertrophy-driven normal postnatal pancreas growth in mice.^29^ As IGS causes chronic hyperaminoacidemia and pancreas expansion, we investigated the underlying cellular and molecular mechanisms. We found that both proliferation and hypertrophy of acinar cells contribute to IGS-induced pancreas expansion and require mTORC1 activation. In addition, SLC38A5 and YAP1 are essential for IGS-induced acinar cell proliferation and hypertrophy (Figure 6F).

Hyperaminoacidemia is the driver of the exocrine pancreas growth in the IGS models. This is supported by abolishment of pancreas expansion in the *Gcgr*^*hep−/−*^ mice by normalizing aminoacidemia using a LPD. It is further supported by the requirement of Slc38a5b for both hypertrophy and hyperplasia of zebrafish acinar cells. *Slc38a5*, the most abundant aaT in WT acinar cells, encodes a low-affinity Na^+^/H^+^ exchange coupled transporter for neutral aa, including the most abundant blood aa glutamine (Km = 3.2 mM) and alanine (Km = 2.5 mM).^50^ Therefore, aa uptake by SLC38A5 is low during normal aminoacidemia as the concentration of these aa is more than 5-fold lower than the Km. In IGS conditions, however, the consequent hyperaminoacidemia increases the influx of neutral aa, which in turn promotes the intake of other aa via aa harmonizers.^50^ Increased intracellular aa and their metabolism likely activate mTORC1 and biosynthesis necessary for acinar cell hypertrophy and proliferation expansion.^15^^,^^20^

Accumulating evidence indicates the presence of endocrine-exocrine crosstalk in the pancreas.^48^ For example, individuals with type 1 diabetes and their first-degree relatives with autoantibodies have a smaller pancreas likely due to few acinar cells.^49^^,^^50^^,^^51^ This may result from reduced insulin secretion by beta cells, leading to reduced insulin action on acinar cells.^52^^,^^53^ Also, insulin signaling in acinar cells protects them from Ca^2+^ overload during acute pancreatitis.^53^ However, our results exclude a direct role of glucagon signaling in suppressing acinar cell hyperplasia and hypertrophy since they persist in *Gcgr*^*hep−/−*^ mice. These mice should have stronger glucagon signaling in acinar cells due to hyperglucagonemia.^19^ The requirement of Slc38a5 and mTORC1 for acinar cell hyperplasia and hypertrophy supports a role for glucagon signaling in determining acinar cell mass by regulating blood aa levels. However, as *Slc38a5* and mTORC1 are also necessary for alpha cell hyperplasia, future studies are needed to determine whether acinar cell proliferation and hypertrophy depend on α cell hyperplasia.

We show that YAP1 is necessary for IGS-induced growth of the exocrine pancreas. Suppression of YAP activity is essential for proper acinar cell differentiation.^44^^,^^45^ Yet, YAP activation is necessary for postnatal regeneration and homeostasis of acinar cells.^46^^,^^51^ For example, in adult mouse pancreas YAP is active in a rare population of Tert^+^ acinar cells that are capable of clonal expansion during homeostasis and after pancreatic injuries.^46^^,^^51^ YAP is necessary for the injury-induced proliferation of these Tert^+^ acinar cells.^46^ Since multiple pathways can activate YAP and only a small fraction of acinar cells have YAP1 activation in IGS mice, future single-cell studies are necessary to understand its activation mechanism in conditions such as hyperaminoacidemia. In zebrafish, Yap activation in acinar cells requires Slc38a5b and mTORC1. mTORC1 can regulate YAP stability through autophagy.^52^ Intriguingly, we found that Yap1 is necessary for mTORC1 activation in acinar cells. YAP/TAZ can stimulate mTORC1 activity via multiple mechanisms.^21^^,^^53^^,^^54^^,^^55^^,^^56^ We propose that the IGS-induced pancreas expansion results from the synergism between mTORC1 and YAP/TAZ signaling pathways (Figure 6F).

YAP1 activation has been reported to cause pancreatitis in mice,^57^^,^^58^ which was not seen in IGS mice. This may be due to the difference in the extent and intensity of YAP1 activation in these models. While previous results were derived from mice with pan-acinar cell YAP1 activation by deleting its upstream suppressors, YAP1 is only weakly activated in a small fraction of acinar cells in our models.

We show both hyperplasia and cell hypertrophy contribute to the IGS-induced expansion of the exocrine pancreas. This is similar to protein-rich diet-induced adaptive pancreas growth in rodents.^8^^,^^10^ However, there are several differences. First, the protein-rich diet exerts a faster response, plateauing within 7–14 days,^9^^,^^10^ while GCGR-Ab caused continuous growth of the exocrine pancreas for the entire 8-week treatment. Second, increased CCK is responsible for high protein diet-induced acinar cell proliferation,^8^ while blood CCK levels were not changed in mice with IGS. Lastly, mTORC1 is only necessary for acinar cell hypertrophy in diet-induced pancreas expansion. In contrast, mTORC1 is required for both proliferation and hypertrophy of acinar cells in IGS models. These differences are likely due, at least in part, to the difference in duration and composition of elevated blood aa in these models. While the high protein diet increases blood aa levels more than 2-fold initially, the levels gradually return to normal within 2 weeks, except for branched chain aa.^59^ In contrast, hyperaminoacidemia is maintained as long as glucagon signaling is disrupted and may result in higher and more persistent mTORC1 activation.

Mechanistic understanding of exocrine pancreas expansion has the potential to improve the treatment of exocrine pancreas insufficiency (EPI). Whether caused by chronic pancreatitis, cystic fibrosis, or other congenital defects, EPI requires lifelong pancreatic enzyme replacement therapy (PERT) dosed with every meal, a therapy that is both expensive and difficult to maintain. *In situ* expansion of acinar cells could reduce patient dependence on PERT.^60^ While pathways that stimulate acinar cell expansion could also increase the risk of pancreatic adenocarcinoma, an increased incidence of acinar cell cancers has not been reported in *GCGR*-deficient patients and animal models.^17^^,^^18^ Further research is needed to determine if hyperaminoacidemia-induced pancreas expansion is a viable method to improve exocrine function in EPI.

In summary, the IGS-induced pancreas expansion results from hyperaminoacidemia-induced acinar cell hyperplasia and hypertrophy. In addition to mTORC1, we identified two new components, Slc38a5 and Yap1, that are necessary for pancreas expansion in zebrafish. Although YAP/TAZ is well-known for its role in organ size control,^38^ its role in postnatal acinar cell growth in response to hyperaminoacidemia has not been described previously. Identification of these signaling pathways in IGS-induced pancreas expansion provides targets for therapeutic control of acinar cell proliferation and hypertrophy.

### Limitations of the study

Our studies have several limitations. Although both mouse and zebrafish IGS models showed acinar cell hyperplasia and hypertrophy, diet manipulation was only done in a mouse model, and genetic manipulation of *slc38a5* and *yap1* was only performed in zebrafish. In addition, the knockdown of *slc38a5* and *yap1* was global, precluding the distinction between a cell-autonomous role and a non-cell autonomous role for these genes. Given the observed difference in Yap1 expression in zebrafish and mouse acinar cells and the stage difference between zebrafish and mice used in the study, the cross-species extrapolation, particularly to humans, should be considered preliminary and needs validation. Additional studies are also required to further clarify the mechanism of mTORC1 and YAP mutual activation in zebrafish acinar cells and whether such mutual regulation is conserved.

## Resource availability

### Lead contact

Further information and requests for resources should be directed to and will be fulfilled by the lead contact, Wenbiao Chen (wenbiao.chen@vanderbilt.edu).

### Materials availability

Materials availability will be available upon request from the lead contact.

### Data and code availability

• All data reported in this paper will be shared by the lead contact upon reasonable request. The RNA-seq data have been deposited in the GEO repository and are publicly available as of the date of publication. Accession numbers are listed in the key resources table.
• This paper does not report any original code.
• Any additional information required to reanalyze the data reported in this paper is available from the lead contact.

## Acknowledgments

This research was supported by R01DK117147 (to W.C. and A.C.P.), R01DK132669 (to E.D.D.), DK106755 (to A.C.P.), and the 10.13039/100000738Department of Veterans Affairs (BX000666, A.C.P.). This research was supported by resources provided by the 10.13039/100020526Vanderbilt Diabetes Research and Training Center (DK020593), including its Islet and Pancreas Analysis Core, Hormone Assay and Analytical Services Core, and Animal Metabolic Physiology Core. We thank the following colleagues for generously sharing mice: Drs. Seung Kim (*Gcg*^*−/−*^), Daniel Drucker (*Gcgr*^hep−/−^ and *Gcgr*^flox^), and Julio Ayala (*Glp1r*^*−/−*^). Experiments were performed in part through the use of the Vanderbilt Cell Imaging Shared Resource, the 10.13039/100020526Vanderbilt Diabetes Research and Training Center, the Islet Procurement and Analysis Core (supported by 10.13039/100000002NIH grants CA68485, DK20593, DK58404, DK59637, and EY08126) and the Vanderbilt Zebrafish Aquatic Facility.

## Author contributions

Conceptualization: A.C.P., C.D., E.D.D., and W.C.; investigation: C.D., Y.Z., Y.G., A.B., Z.T., K.S., E.S., B.A.C., J.S., R.J., T.M.R., and R.A.B.; writing—original draft: C.D., Y.G., and W.C.; writing—review and editing: A.C.P., E.D.D., D.C.S., J.J.W., and M.B.; visualization: C.D., Y.Z., Y.G., D.C.S., K.S., S.S., K.S., and B.A.C.; funding acquisition: A.C.P., E.D.D., and W.C.

## Declaration of interests

The authors declare no competing interests.

## STAR★Methods

### Key resources table

**Table:** 

REAGENT or RESOURCE	SOURCE	IDENTIFIER
**Antibodies**		

anti-alcam	DSHB	Cat# ZN-8
anti-Chicken IgY (H+L) - Alexa Fluor® 488	Invitrogen	Cat# A-11039; RRID: AB_2534096
anti-GFP	Aves	Cat# GFP-1010; RRID: AB_2307313
anti-mCherry	Novus Biologiclas	Cat# NBP2-25157; RRID: AB_2753204
anti-MHC	DSHB	Cat# MF20
anti-mouse IgG1 - Alexa Fluor® 488	Invitrogen	Cat# A-21121; RRID: AB_2535764
anti-mouse IgG2b - Alexa Fluor® 568	Invitrogen	Cat# A-21144; RRID: AB_2535780
anti-rabbit (H+L) Superclonal™ - Alexa Fluor® 647	Invitrogen	Cat# A-27040; RRID: AB_2536101

**Bacterial and virus strains**		

DH5-alpha competent E.coli	New England Biolabs	Cat# C2987I

**Chemicals, peptides, and recombinant proteins**		

1-phenyl-2-thiourea (PTU)	Sigma-Aldrich	Cat# P7629-10
16% Paraformaldehyde aqueous solution	Electron Microscopy Sciences	Cat# 15710
2,3-Butanedione monoxime	Sigma-Aldrich	Cat# B0753
4-hydroxytamoxifen	Sigma-Aldrich	Cat# H7904
4’,6-Diamidino-2-phenylindole (DAPI)	Sigma-Aldrich	Cat# D9542
Acetone	Sigma-Aldrich	Cat# 320110
BODIPY™ FL C5-Ceramide	Invitrogen	Cat# D3521
BSA	Sigma-Aldrich	Cat# A7906
Chloroquine diphosphate salt	Sigma-Aldrich	Cat# C6628
Chromium Nuclei Isolation Kit with RNase Inhibitor	10x Genomics	Cat# PN-1000494
CRISPR-Cas9 tracrRNA	IDT	Cat# 1072534
Dimethyl sulfoxide	Sigma-Aldrich	Cat# D4540
DNA Clean & Concentrator	Zymo Research	Cat# D4014
Epon	Sigma-Aldrich	Cat# 45359
Ethanol	Grogg Chemie	Cat# G003
Fast Digerst HindIII	Thermofisher Scientific	Cat# FD0504
Fast Digest BamHI	Thermofisher Scientific	Cat# FD0054
Fast Digest ScaI	Thermofisher Scientific	Cat# FD0434
foetal bovine serum	Sigma-Aldrich	Cat# F7524
Fragment Analyzer NGS Fragment Kit	Agilent	Cat# DNF-473
Gateway LR Clonase II Enzyme mix	Invitrogen	Cat# 11791020
Glutaraldehyde	Agar Scientific	Cat# AGR1009
Goat serum	Dominique Dutscher	Cat# S2000
HiFi Cas9 Nuclease V3	IDT	Cat# 1081060
illumina NovaSeq 6000 S1 Reagent Kit v1.5	Illumina	Cat# 20028319
iScript Reverse Transcription SuperMix	Bio-Rad	Cat# 1708841
KCl	Sigma-Aldrich	Cat# P9333
Leibovitz's L-15 Medium	Thermo Fisher Scientific	Cat# 11415064
LysoTracker™ Deep Red	Invitrogen	Cat# L12492
Maxima First Strand cDNA synthesis kit	Thermo Fisher Scientific	Cat# K1671
OsO4	Electron Microscopy Sciences	Cat# 19100
*pENTR/D-TOPO vector*	Invitrogen	Cat# K240020
Phosphate buffered saline	NZYtech	Cat# MB18201
PowerUp SYBR Green Master Mix	Thermo Fisher Scientific	Cat# A25742
Prep User Guide	10X Genomics	Cat# CG000505
Proteinase K	Roche	Cat# 03 115 801 001
Q5® High-Fidelity DNA Polymerase	New England Biolabs	Cat# M0491
Qubit dsDNA HS Assay Kit	Thermo Fisher Scientific	Cat# Q32851
Sodium cacodylate trihydrate	Sigma-Aldrich	Cat# C0250
T7 Endonuclease I	New England Biolabs	Cat# M0302
Triton X-100	Sigma-Aldrich	Cat# T9284
TRIzol Reagent	Invitrogen	Cat# 10296010

**Deposited data**		

snRNASeq Data	NCBI GEO	GEO: GSE246850
Raw data	Zenodo	Zenodo: https://doi.org/10.5281/zenodo.13982794

**Experimental models: Transgenic zebrafish models used in the study**		

Tg(*actb2:mRFP-GFP-map1lc3b*)^*udc2Tg*^	Allende lab	ZDB-ALT-210122-18
TgKI(*mRFP-map1lc3b*)^*brn7*^	This study, Mercader lab	ZDB-ALT-230926-15
Tg*(CMV:EGFP-map1lc3b)*^*zf155*^	Kishi lab	ZDB-ALT-091029-2
TgBAC*(lamp2:RFP)*^*pd1117*^	Affolter lab	ZDB-ALT-150520-1
Tg(*fli1a:GFP*)^*y1*^	ZIRC	ZDB-ALT-011017-8
Tg*(fli1a:DsRedex)*^*um13*^	Lawson lab	ZDB-ALT-100525-3
Tg*(kdrl:GFP)*^*la116*^	Stainier lab	ZDB-ALT-070529-1
Tg*(fli1a:Gal4FF)*^*ubs3*^	Affolter Lab	ZDB-ALT-120113-6
Tg*(kdrl:EGFP-CAAX)*^*ubs47*^	Affolter lab^100^	
Tg*(myl7:GFP)*^*f1*^	Djonov lab	ZDB-ALT-060719-2
Tg*(myl7:mCherry)*^*ko08*^	Kawahara lab	ZDB-ALT-090423-3
Tg*(EPV.Tp1-Mmu.Hbb:CreERT2,cryaa:mCherry)*^*s959*^	Singh lab	ZDB-ALT-131001-3
Tg*(–3.5ubi:loxP-EGFP-loxP-mCherry)*^*cy1701*^	Zon lab	ZDB-ALT-110124-1
*nrs* (^*spns1hi891Tg/hi891Tg*^*)*	Kishi lab	ZDB-FISH-150901-8505
Tg*(UAS:spns1)*^*brn8*^	This study, Mercader lab	ZDB-ALT-230926-16
Tg*(UAS:myc-Notch1-intra)*^*kca3Tg*^	ZIRC	ZDB-ALT-020918-8

**Oligonucleotides**		

See Table S7.		

**Plasmids**		

*pKHR4*	Addgene	Cat# 74592; RRID: Addgene_74592
pDestTol2pA2CrymCherry	Addgene	Cat# 64023; RRID: Addgene_64023

**Software and algorithms**		

Code and analyses	This study	https://github.com/MercaderLabAnatomy/PUB_Chavez_et_al_2023
Fiji	https://fiji.sc/	https://doi.org/10.1038/nmeth.2019
Matlab	https://ch.mathworks.com/products/matlab.html	R2024a Update 3
Napari	https://napari.org	https://doi.org/10.5281/zenodo.3555620
R v4.0	https://www.r-project.org/	R version 4.0.0
Seurat v4.0	CRAN v4.0	https://doi.org/10.1016/j.cell.2021.04.048
T-MIDAS	https://github.com/MercaderLabAnatomy/T-MIDAS	https://doi.org/10.5281/zenodo.10728503

### Experimental model and study participant details

#### Mice

Mice were housed under a 12-h light/12-h dark cycle in individually ventilated cages with automatic water and *ad libitum* access to standard rodent chow (with 24.1% protein) at Vanderbilt animal facilities. The cages had bedding with paper rolls for nesting. Some cages had refuges (huts). All experiments were conducted according to protocols and guidelines approved by the Vanderbilt University Institutional Animal Care and Use Committee. C57BL/J6 male mice and *Gcg*−/− (GKO) mice in *NOD.Cg-Prkdc*^*scid*^ *Il2rg*^*tm1Wjl*^*/SzJ* (NSG) background were obtained from The Jackson Laboratory and bred at Vanderbilt.^25^^,^^61^ *Glp1r*−/− mice in C57BL/J6 background were provided by Dr. Julio E Ayala (Vanderbilt University).^62^ *Gcgr*^*flox*^ and *Gcgr*^*hep−/−*^ mice in C57BL/J6 background have been characterized previously (Figure S1).^19^ They were provided by Dr. Daniel Drucker initially was were bred at Vanderbilt. To pharmacologically interrupt glucagon signaling, C57BL/J6 mice were intraperitoneally injected with 10 mg/kg of GCGR neutralizing monoclonal antibody (GCGR-Ab) or IgG from Eli Lilly once a week for 2, 4, or 8 weeks. To be consistent with our prior study, only male mice were treated.^63^ Sirolimus (SIR, rapamycin, NDC 0008-1030-06, Pfizer) or PBS was given by intraperitoneal (i.p.) injection every 72 h at 1.5 mg/kg^64^ To correct hyperaminoacidemia, *Gcgr*^*flox*^ and *Gcgr*^*hep−/−*^ male and female mice (3–40 weeks old) along with age-matched control littermates were fed with isocaloric normal (20%) and low (6%) protein diets (Harlan Teklad #TD.91352 and #TD.90016, respectively). For AA measurement, serum samples were collected during the daytime from GKO and control mice at 14- to 20-week-old, from IgG or GCGR-Ab treated C57BL/J6 and *Glp1r*^*−/−*^ mice after 8 weeks of treatment, or from *Gcgr*^*flox*^ and *Gcgr*^*hep−/−*^ mice at the end of experiments (Table S3).

#### Zebrafish

Zebrafish were raised at 27°C in an Aquatic-Habitats system and embryos were raised at 28.5°C in an incubator on a 14/10-h light/dark cycle. The age of zebrafish was expressed as days postfertilization (dpf). As larval zebrafish cannot be distinguished by sex, both males and females were used. The *gcgr*−/− zebrafish (*gcgra*−/−;*gcgrb*−/−) were described previously (Table S3).^26^ Drugs were administered in the medium.

### Method details

#### Mice

##### Serum measurements

Mice were fasted for 6 h with free access to water, and blood was collected from the retro-orbital sinus. Aprotinin protease inhibitor (PentaPharm) was pre-added to collection tubes to yield a final concentration in whole blood of 1000KIU. The serum was collected and stored at −80^o^C until analysis. CCK and GLP-1 measurements were performed using CCK Enzyme Immunoassay kit (RayBio, #EIA-CCK) and Total GLP-1 NL-ELISA kit (Mercodia, 10-1278-01). Total serum amino acid levels were measured using an L-Amino Acid Quantification kit (Sigma MAK002). Individual amino acid levels were quantified by HPLC as described previously.^20^

##### Immunofluorescence and imaging

Pancreata were fixed, sectioned, and stained as previously described.^65^^,^^66^^,^^67^ Primary antibodies used in this project are listed in the key resources table. Apoptosis was assessed by TUNEL (Millipore, S7165) following the manufacturer’s instructions. Nuclei were stained with Hoechst 33342 (0.5 μg/mL, Thermo Fisher Scientific) or DAPI (300 nM, Thermo Fisher Scientific). Images were acquired with a fluorescence ScanScope (Aperio) scanner or a confocal laser-scanning microscope (LSM880, Carl Zeiss, Jena, Germany). Positive Ki67 acinar cells were quantified, and cell size was measured using HALO image analysis (Indica Labs). pS6 intensity was quantified using ImageJ.

##### Acinar cells RNA isolation, cDNA synthesis, quantitative RT-PCR, and RNA-seq

To purify acinar cells, the mouse pancreas was digested by collagenase and acini were picked by hand.^68^ Total RNA was extracted from acinar cells using an RNAqueous RNA isolation kit (Ambion, Austin, TX). RNA quality control and quantity assessment (QC/QA) was performed using a Bioanalyzer instrument. The average RNA integrated Number (RIN) was 7.8 + 0.09 (IgG, 7.5–8.1) and 8.0 + 0.14 (GCGR-Ab, 7.4–8.4). cDNA was synthesized using High Capacity cDNA Reverse Transcription Kit (Applied Biosystems, 4368814) according to the manufacturer’s instructions. Quantitative PCR (qPCR) was performed using TaqMan assays (key resources table) with reagents from Applied Biosystems (Foster City, CA) as previously described.^66^^,^^69^^,^^70^ *Actb* was used for normalization. Relative changes in mRNA expression were calculated by the comparative ΔCt method.

RNA-seq was performed by MEDGENOME (Foster City, CA). About 72–137 million uniquely mapped reads were acquired per sample. Alignment was performed using STAR (v2.7.3a) aligner to the reference mouse genome (genome-build GRCm38.p6).^71^ The raw read counts were estimated using HTSeq (v0.11.2). Further quality control and downstream analysis were performed in the Strand NGS analysis platform v3.4 (Strand life Sciences) where read counts were normalized using TMM (Trimmed Mean of M values).^72^ Genes with less than 20 counts across samples were removed.

##### Relative pancreas weight measurement

To determine relative pancreas weight, mice were anesthetized, weighed, and the pancreas was carefully dissected and placed into a 10 cm Petri dish containing ice-cold PBS. After removing fat tissue, the pancreas was blotted with filter paper and weighed to determine the absolute pancreas weight. The weight was normalized to body weight and described as relative pancreas weight.

#### Zebrafish

##### Mutagenesis

The knockdown of *slc38a5b*, *taz*, and *yap* was performed according to Yin et al.^73^ The mutagenesis was initially determined by the heteroduplex motility assay. PCR products of the targeted region from pools of control and mutagenized embryos were subjected to Sanger sequencing and Synthego ICE or TIDE^74^ analysis to determine the mutagenesis rate (Table S2).

##### Pancreas area, volume, and acinar cell proliferation analysis

Because zebrafish pancreas is difficult to remove and weigh accurately, the pancreas size was quantified as pancreas area or pancreas volume by confocal imaging of fixed *Tg(ela3l:EGFP)* zebrafish.^27^ Proliferation by 24-h EdU labeling was done as previously described.^14^^,^^26^ Some fish were treated with rapamycin (200 nM) for 24 h before euthanasia. Fish were euthanized by MS-222 and fixed in 4% paraformaldehyde (PFA), equilibrated in 20% sucrose and embedded into Optimal Cutting Temperature (OCT) media to generate pancreas-containing cryosections (12 μm). EdU was detected using the Click-iT EdU Alexa Fluor 594 Imaging Kit (C10339; Invitrogen).

Acinar cells were labeled using an amylase antibody (Rabbit, Sigma, A8273). Acinar cell size was measured in *Tg(ela3l:EGFP)-*carrying zebrafish by dividing the GFP+ area with the number of DAPI stained nuclei. All images were collected using Zeiss LSM880 (Carl Zeiss, Jena, Germany) and analyzed by Imaris (Oxford Instruments).

### Quantification and statistical analysis

Statistically significant differences were determined by two-tailed t-tests (2 groups) or one-way ANOVA (analysis of variance) followed by Tukey’s Multiple Comparisons Tests (>2 groups). A *p* value < 0.05 was considered statistically significant. Values reported represent mean ± SEM. In RNA-seq analysis, *p*-values were estimated using Z-test (Strand NGS) for differential expression. False discovery rate adjusted for multiple hypothesis testing with Benjamini-Hochberg (BH) procedure, where *p*-value <0.05 and fold change ≥1.5 were used to define differentially expressed genes. Differentially expressed genes were further analyzed through Ingenuity Pathway Analysis (IPA, Qiagen) and Gene Ontology (GO) analysis using DAVID v6.8.^75^
