# Supplementary material for: Hyperaminoacidemia from interrupted glucagon signaling increases pancreatic acinar cell proliferation and size via mTORC1 and YAP pathways
Source: iScience. 2024 Nov 22;27(12):111447. doi: 10.1016/j.isci.2024.111447 (PMC11667045; doi:10.1016/j.isci.2024.111447)
Supplement: Document S1. Figures S1–S11 and Tables S1–S3 [file mmc1.pdf]

iScience, Volume *27*

Supplemental information

Hyperaminoacidemia from interrupted glucagon signaling increases pancreatic acinar cell proliferation and size via mTORC1 and YAP pathways

Chunhua Dai, Yue Zhang, Yulong Gong, Amber Bradley, Zihan Tang, Katelyn Sellick, Shristi Shrestha, Erick Spears, Brittney A. Covington, Jade Stanley, Regina Jenkins, Tiffany M. Richardson, Rebekah A. Brantley, Katie Coate, Diane C. Saunders, Jordan J. Wright, Marcela Brissova, E. Danielle Dean, Alvin C. Powers, and Wenbiao Chen

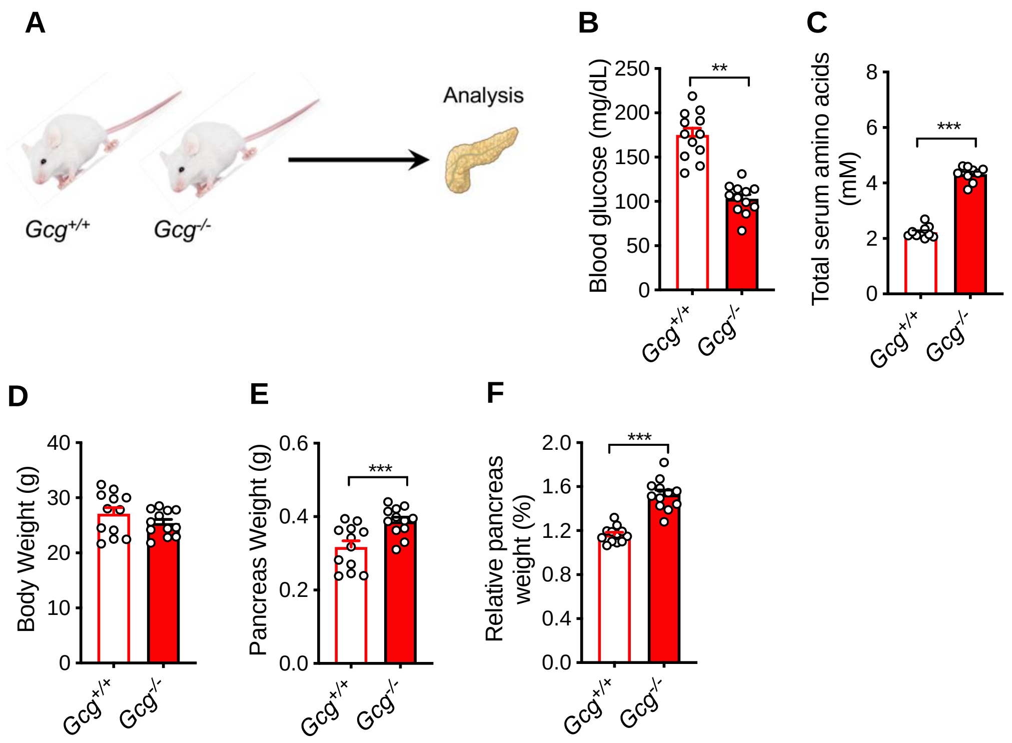

Figure S1. Interruption of glucagon signaling increases total amino acid levels in blood and pancreas weight in *Gcg$^{-/-}$* mouse model. (A) Model 1, Gcg$^{+/+}$ and Gcg$^{-/-}$ mice. (B) Random blood glucose; (C) Total serum amino acid levels; (D) Body weight, (E) Absolute pancreas weight; (F) Relative pancreas weight (% of abosolute pancreas weight/body weight). N=12 mice/group. * p<0.05, ** p<0.01, *** p<0.001. Data represent means $\pm$ SEM in all figures.

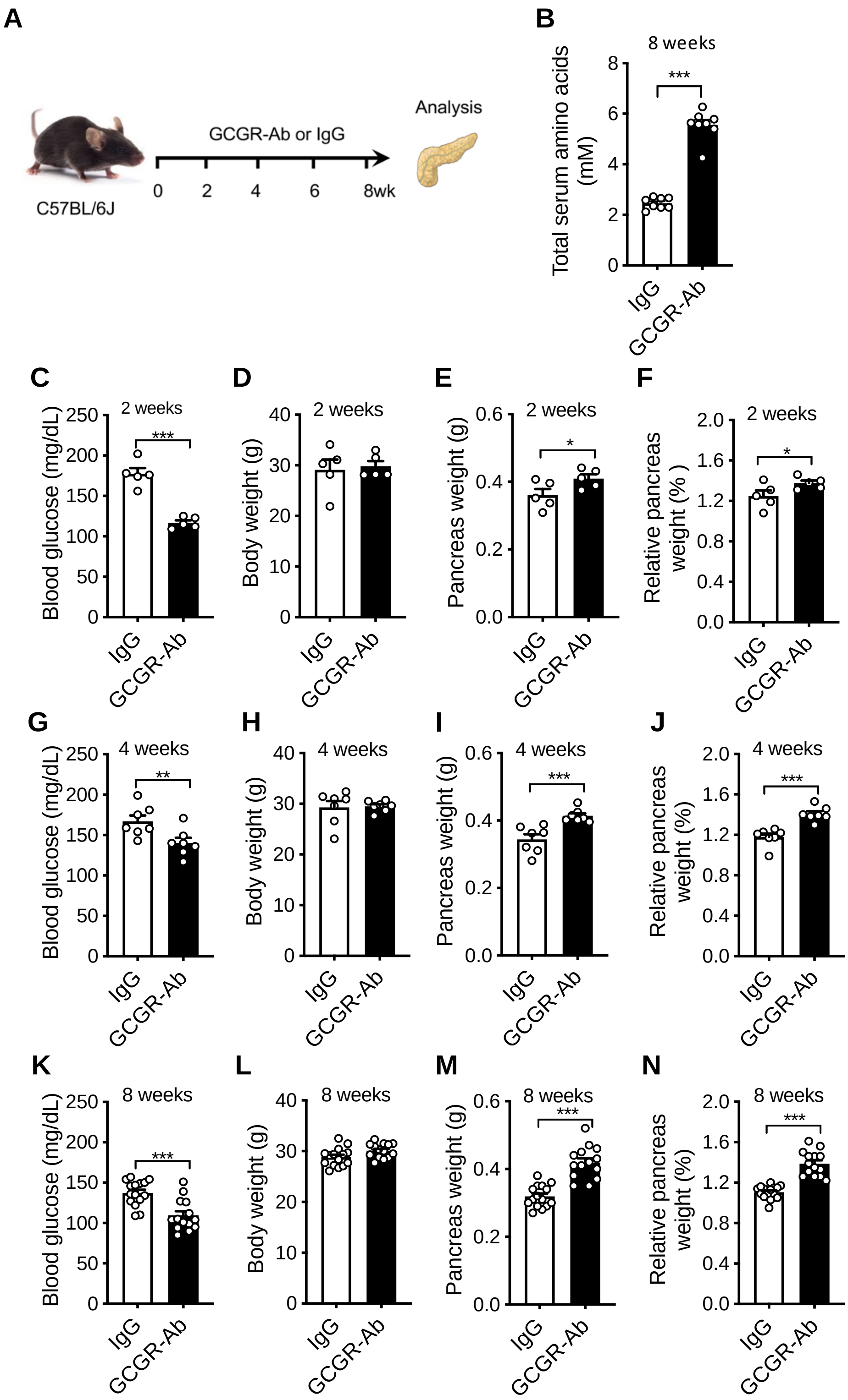

Figure S2. Interruption of glucagon signaling increases total amino acid levels in blood and pancreas weight in mice treated with GCGR-Ab. (A) Model 2, C57BL/6J mice treated with GGR-Ab to block glucagon signaling. (B) Total serum amino acid levels in blood; (C-F) 2 weeks treatment; (G-J) 4 weeks treatment; (K-N) 8 weeks treatment; (C,G,K) Blood glucose; (D,H,L) Body weight; (E,I,M) Absolute pancreas weight; (F,J,N) Relative pancreas weight (% of absolute pancreas weight/body weight). N=6-14 mice/group * p<0.05, ** p<0.01, *** p<0.001

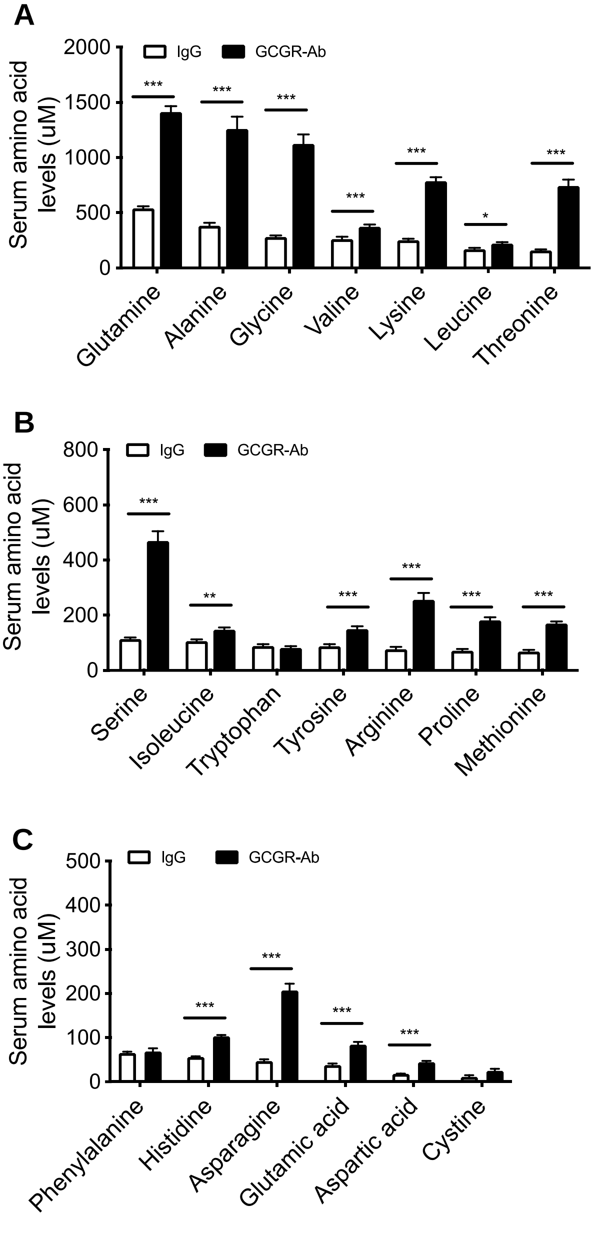

Figure S3. GCGR-Ab treatment increases most amino acids in blood (AAs). (A) high concentration AAs; (B) medium concentration AAs; (C) low concentration AAs. N=8 mice/group. * p<0.05, ** p<0.01, *** p<0.001

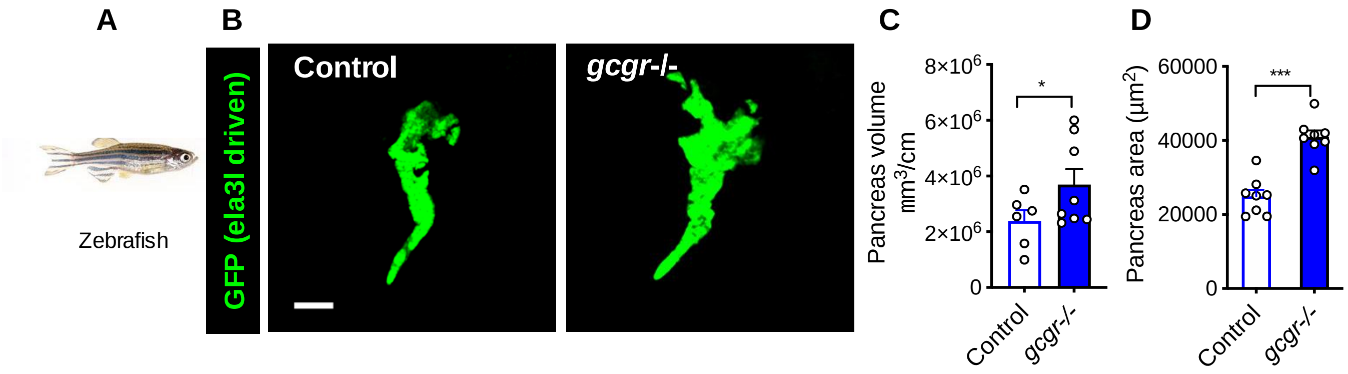

Figure S4. Interruption of glucagon signaling increases pancreas mass in *Gcgr*$^{-/-}$ zebrafish. (A) Model 3, *gcgr*$^{+/+}$ (control) and *gcgr*$^{-/-}$ zebrafish. (B) Representative of pancreas images from *ela3l/*:GFP fish at 18 dpf; (C,D) Pancreas volume and area in gcgr-/- and control zebrafish. N=6-8/group. * $p<0.05$, ** $p<0.01$, *** $p<0.001$

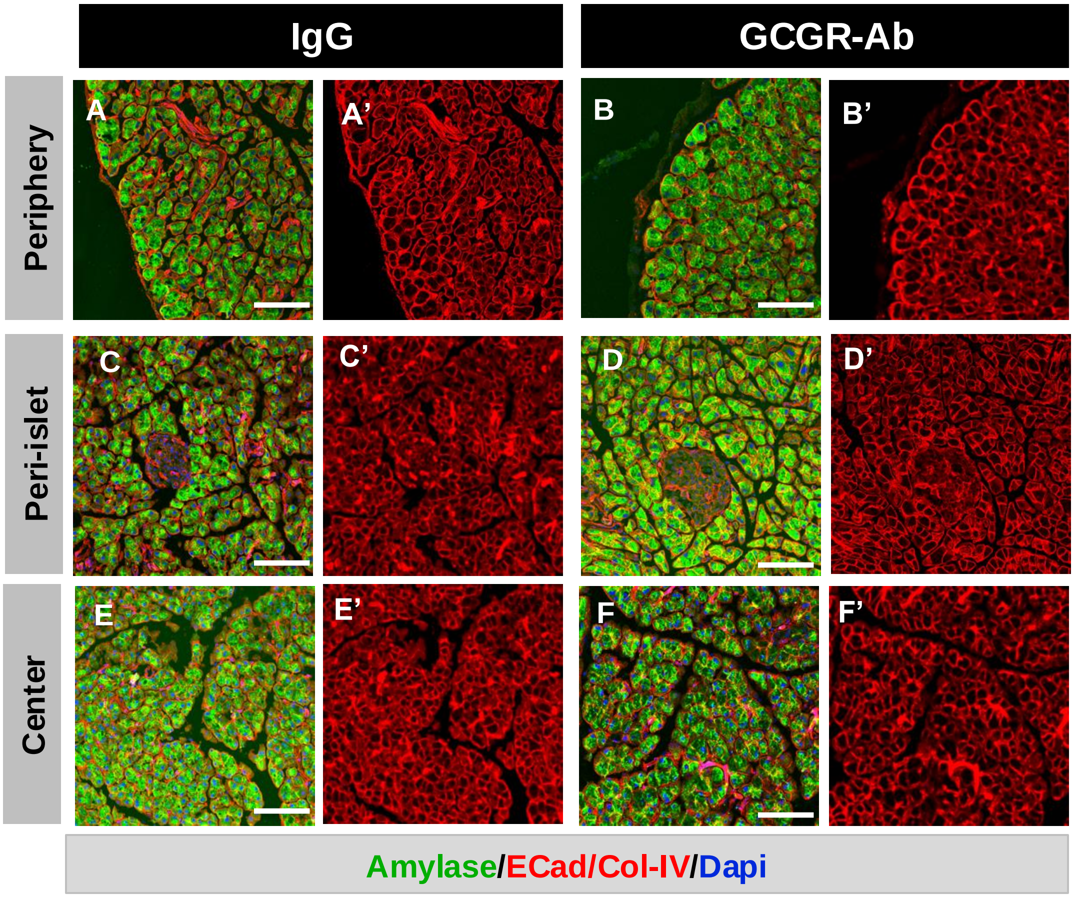

Figure S5.Interruption of glucagon signaling increases acinar cell size in all pancreas regions. Representative immunofluorescence images of acinar cell size from IgG (A,C,E) and GCGR-Ab (B,D,F) treated mice (8 weeks). (A,B) Periphery area, (C,D) Peri-islet area, (E,F) Center region. Green, amylase; red, E-Cadherin+Col-IV (cell border markers); blue, DAPI. (A',B',C',D',E',F') only show cell borders for better viewing of cell size. Scale bar=100 μm.

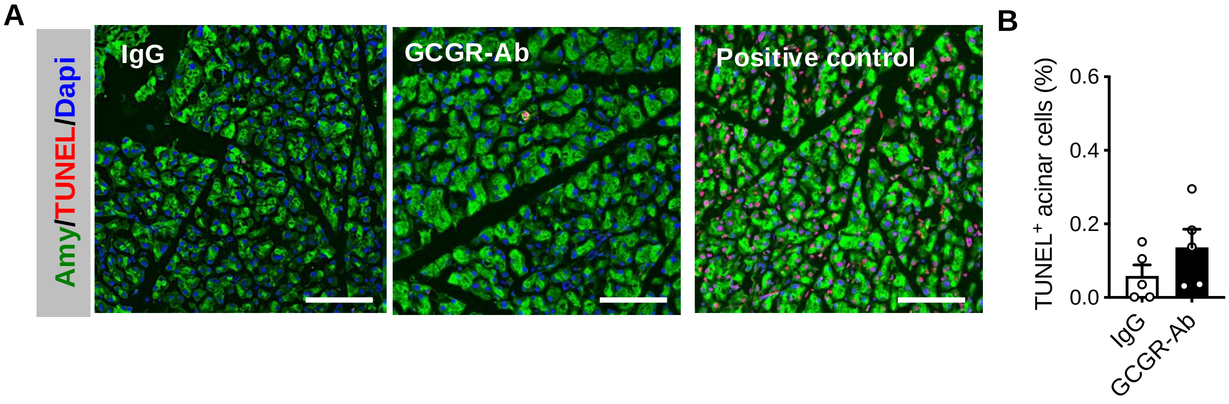

Figure S6. Interruption of glucagon signaling does not induce apoptosis. (A) Representative images of TUNEL assay. DNase I treated samples are included as positive controls. Scale bar=100 μm.(B) Quantification of TUNEL positive acinar cells.

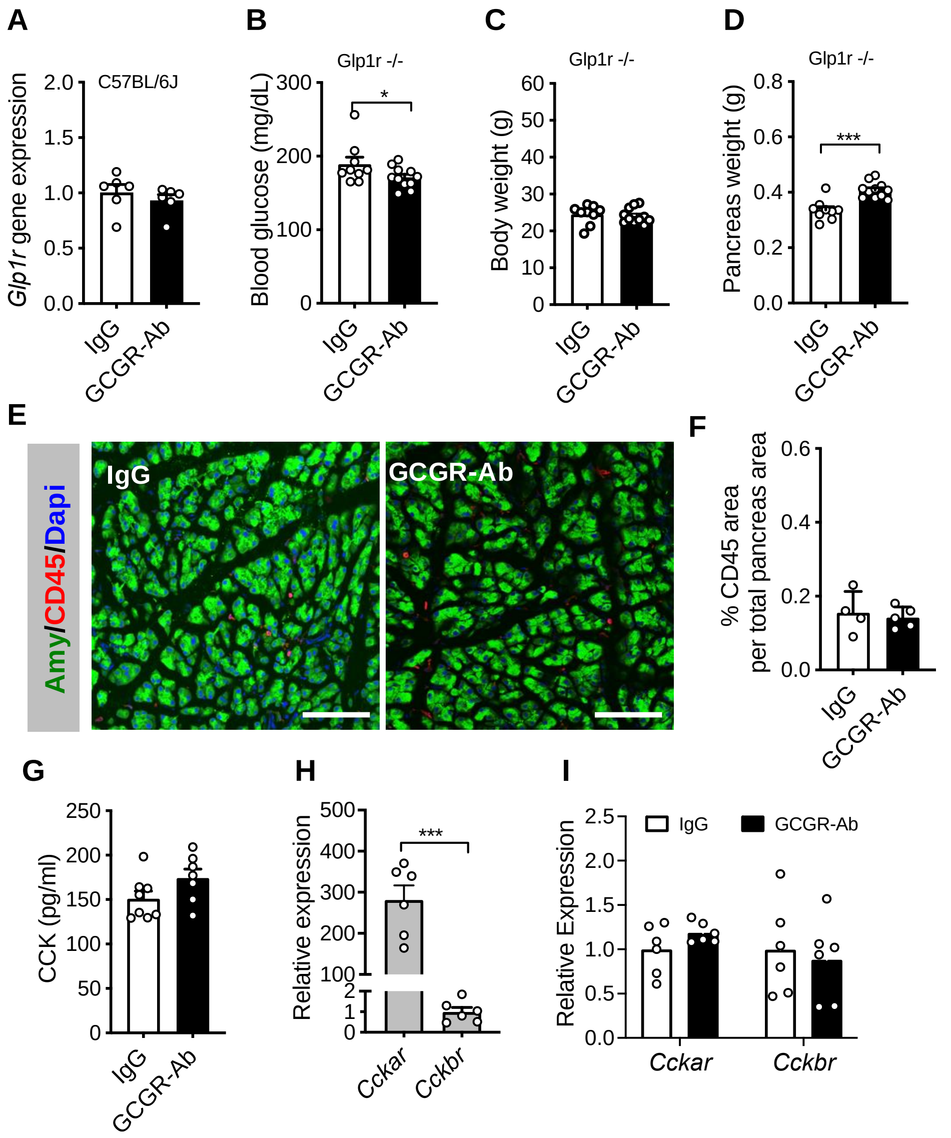

Figure S7. IGS induced pancreas expansion is independent GLP1, pancreatitis, and CCK. (A) Relative expression of *Glp1r* gene in the acinar cells of C57BL/6J mice treated with IgG or GCGR-Ab. N=6/group. (B,C,D) Glucose, body weight, and absolute pancreas weight in the *GLP1*$^{-/-}$ mice treated with GCGR-Ab or IgG for 8 weeks. N=8-14/group. (E) Representative images of CD45 immunofluorescence in IgG or GCGR-Ab treated C57BL/6J pancreas section. Green, amylase; red, CD45; blue, DAPI. Scale bar=100μm. (F) Quantification of CD45 positive area in pancreas sections. N=4-5/group. (G) CCK concentrations in blood in the c57BL/6J mice treated with IgG or GCGR-Ab for 8 weeks. N=6-8/group (H) Relative expressions of *Cckar* and *Cckbr* gene in C57BL/J6 mice. N=6/group (I) Comparison of *Cckar* and *Cckbr* gene expression in C57BL/J6 mice treated with GCGR-Ab. N=6-7/group. * p<0.05, ** p<0.01, *** p<0.001

Figure S8

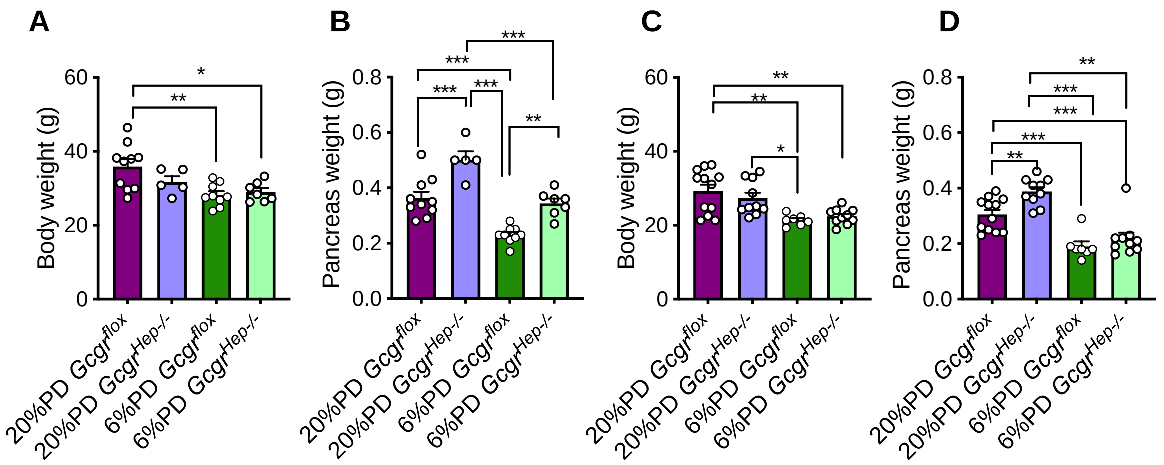

Figure S8. Phenotypes of *Gcgr*(hep)$^{-/-}$ on low protein. (A, B) Body weight and pancreas weight in *Gcgr*(hep)-/- with 20% or 6% protein diet from 22 to 40 weeks of age; (C,D) Body weight and absolute pancreas weight in *Gcgr*(hep)-/- with 20% or 6% protein diet for 3 to 17 weeks of age. N=5-12 mice/group. * p<0.05, ** p<0.01, *** p<0.001

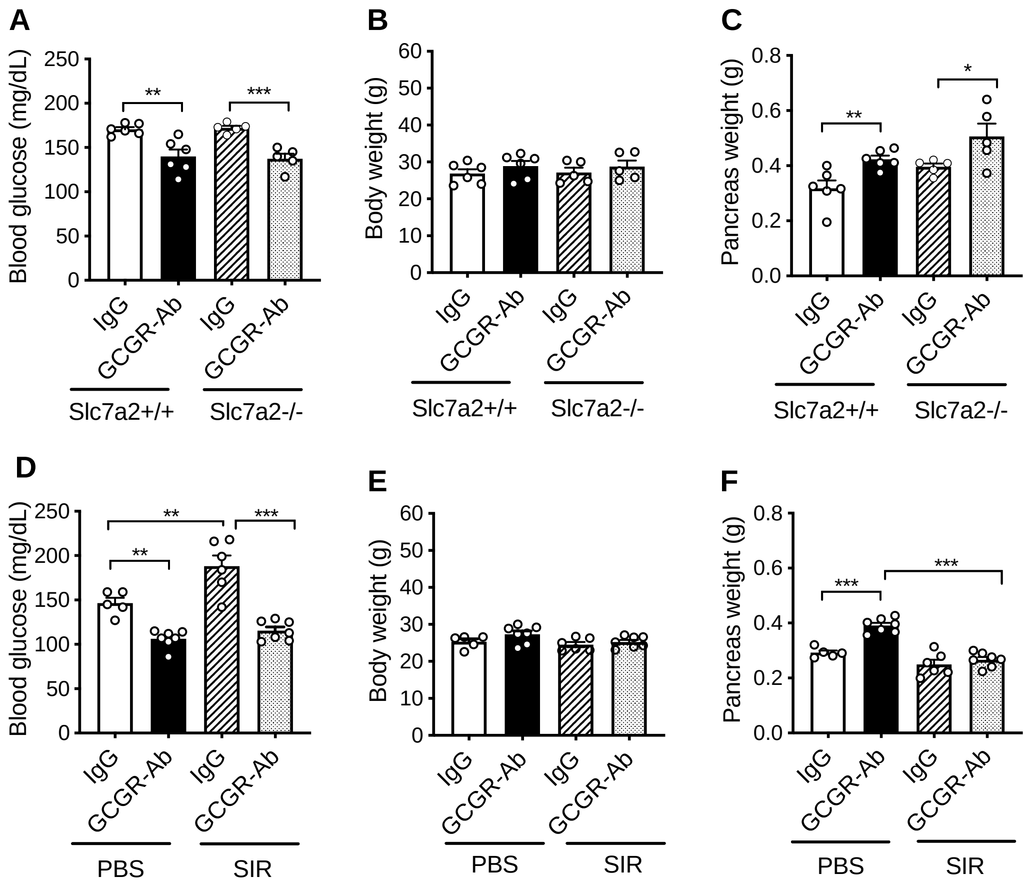

Figure S9. Phenotypes of *Slc7a2* and sirolimus treatment. (A,B,C) *Slc7a2*$^{+/+}$ and *Slc7a2*$^{-/-}$ mice treated with IgG or GCGR-Ab for 8 weeks. N=5-6/group. (D,E,F) C57BL/6J mice treated with IgG or GCGR-Ab and with or without sirolimus for 4 weeks. N=5-6/group. (A,D) Blood glucose; (B,E) Body weight; (C, F) Absolute pancreas weight. * $p<0.05$, ** $p<0.01$, *** $p<0.001$

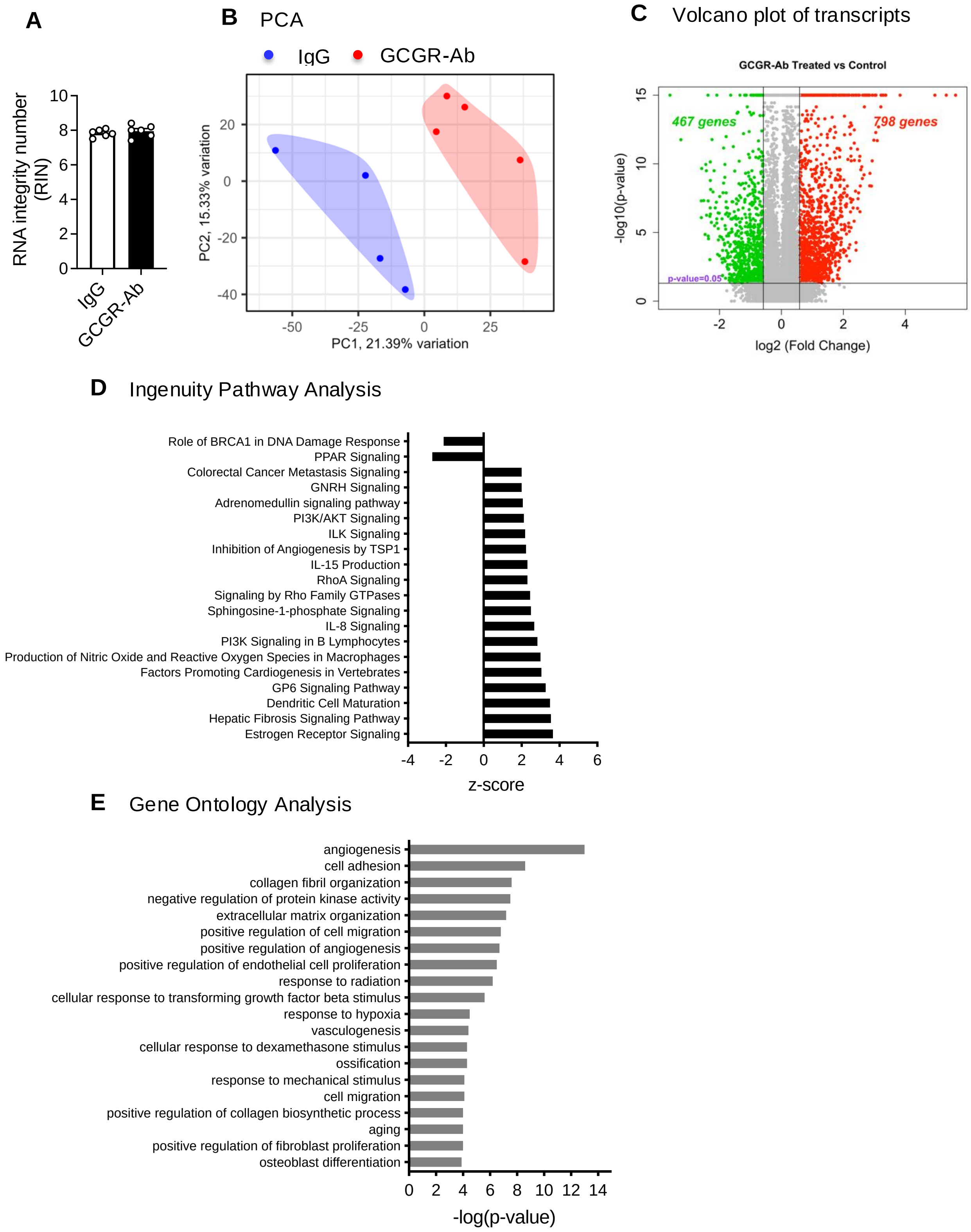

Figure S10. Acinar cell RNAseq analysis. RNAs were isolated from C57BL/6J mice treated with IgG or GCGR-Ab for 8 weeks. (A) RNAs used in this project are high quality (RIN 7.4 to 8.4). (B) Principle component analysis of the RNAseq datasets. (C) Volcano plot of differentially expressed transcripts. (D) Ingenuity pathway analysis. (E) Gene ontology analysis.

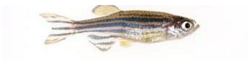

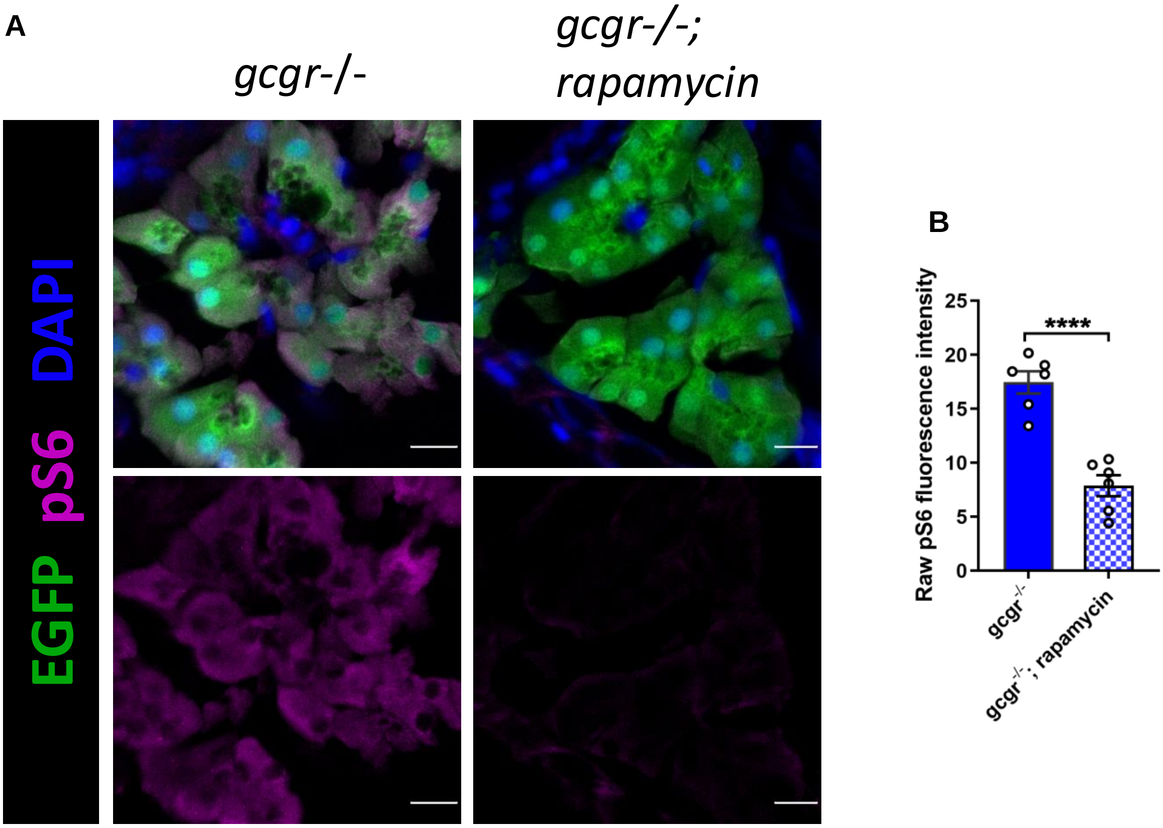

Figure S11. Validation of rapamycin treatment. (A) Representative immunofluorescence images of pS6(240/244) in pancreas sections from fish treated with vehicle or 100 nM rapamycin for 24 hours. All fish carry the Tg(ela3l:EGFP) transgene that labels acinar cells with EGFP (scale bar, 10 μm). (B) Quantification of raw pS6 signal intensity in acinar cells of these fish.

Supplemental Table 1: AA transporter genes in zebrafish acinar cells

Gene Symbol	Normalized Counts
slc1a4	32717.87
slc38a5b	20692.85
slc38a2	9693.06
slc1a3a	5756.60
slc25a22	4856.41
slc43a1b	4634.98
slc3a2b	3214.39
slc7a10a	3066.04
slc3a2a	2590.99
slc16a10	2572.65
slc7a2	2173.45
slc7a3a	2161.57
slc43a1a	1441.10
slc17a7	1165.84
slc38a10	880.20
slc38a4	605.26
slc15a4	568.33
slc38a7	530.70
slc38a3b	384.51
slc3a1	196.00
slc6a20	147.49
slc7a14a	122.55
slc43a2a	85.54
slc36a1	83.94
slc43a2b	82.46
slc7A8b	48.78
slc7a7	30.34
slc38a5a	7.88

Supplemental Table 2: Primers used in CRISPR/Cas9 mutagenesis in zebrafish

**Gene name**		**Target sequence**	**Forward genotyping primer**	Reverse genotyping primer
slc38a5b	sgRNA1	ggACCTCAGCAATGCCATCA	AGTGATGGAGGAAAAACCCCTC	GGGTGATGCATTGTGGGATACT
	sgRNA2	ggCGAGCGTTTGGACACCCG	GCATGTGAAGATTTGTGTCCAT	TACCACCTATGTTGTGCATCGT
yap1	sgRNA1	GGAAGTATCTCTGTCCCGAA	GCCAGTCCTCTTACGAGATACC	AGCAGCATTTCAGACTTAACCC
	sgRNA2	gGGTCCTCTTCCTGACGGGT	TTGGAACAAGTAAAGGGGTGAG	TAAATAGCAGCAACAGCGTCAT
taz	sgRNA1	GgAGGACTGGTGCCGGGTGT	CTCCTGGAGGAACAAGGATATG	GAGAAAGTACTTCTGGCCGTTG
	sgRNA2	GgGATGGCCTTCACCCCCAA	AGACTCCACTCCCACACCC	ACCGTGTTCAATACTCATTCCC

GG at the 5’ end of sgRNA is necessary for efficient transcription by T7 RNA polymerase.
Lower case g indicates a mismatch with genomic DNA.

Supplemental Table 3:IGS models

	Model	Control genotype (if applicable)	Treatment	Blood glucose	CCK	Serum AAs	Pancreas size	Acinar cell size	Acinar cell proliferation	Gene expression	pS6 IHC	Yap IHC
Mouse	*Gcg*$^{-/-}$	*Gcg*$^{+/+}$	-	●		●	●	●	●		●	●
	C57BL/6J	-	IgG/GCGR-Ab	●	●	●	●	●	●	●	●	●
			igG/GCGR-Ab; PBS/SIR				●		●			
	Gcgr$^{hep-/-}$	*Gcgr*^{flox}	20%PD, 6%PD			●	●					
Zebrafish	*gcgr*$^{-/-}$; *tg(ela3l:EGFP)*	*gcgr*$^{+/+}$; *tg(ela3l:EGFP)*	-				●	●	●			
			slc38a5b-sgRNA					●	●			●
			taz-sgRNA					●	●			
			yap-sgRNA					●	●		●	

Ab, antibody; AAs, amino acids; IHC, immunohistochemistry; PD, protein diet; sgRNA, guide RNA; SIR, sirolimus.
